# Genome‐wide identification and solute selectivity of aquaporins in the sharpnose sevengill shark, *Heptranchias perlo*

**DOI:** 10.14814/phy2.70895

**Published:** 2026-04-24

**Authors:** Shinichiro Hidaka, Ayumi Nagashima, Akira Kato

**Affiliations:** ^1^ School of Life Science and Technology Institute of Science Tokyo Yokohama Japan

**Keywords:** Aqp8, aquaglyceroporin, aquaporin, cartilaginous fish, sharpnose sevengill shark

## Abstract

The aquaporin genes (*aqps*) are widely distributed among vertebrates. In cartilaginous fish, *aqp3* and *aqp10* are duplicated, whereas many species have lost *aqp8*. While the solute permeability of aquaglyceroporins and Aqp8 exhibits significant diversity, systematic evaluations of their activity within species are limited. Consequently, we conducted a comprehensive analysis of the *aqp*s in various cartilaginous fish genome databases. Our analysis revealed that the sharpnose sevengill shark (*Heptranchias perlo*) and North Pacific spiny dogfish (*Squalus suckleyi*) possess all *aqp* genes found in cartilaginous fish, rendering them suitable for comparative analysis of Aqp activity. The activity of all aquaglyceroporins and Aqp8 in the sharpnose sevengill shark was analyzed using *Xenopus* oocyte swelling assays. The water permeability of Aqp3c2, Aqp8, Aqp9, Aqp10c1, and Aqp10c2; the glycerol permeability of Aqp3c2, Aqp9, and Aqp10c2; the urea permeability of Aqp3c2, Aqp8, Aqp9, and Aqp10c2; and the boric acid permeability of Aqp8 and Aqp9 were determined. The urea and boric acid permeabilities of Aqp9 were higher than those of other Aqps. Our results provide genomic evidence for the loss of *aqp7* and further elucidate the diversification of *aqp8* in cartilaginous fishes. Moreover, the urea permeability of Aqp9 suggests a functional role in their unique urea‐based osmoregulation strategy.

## 1 INTRODUCTION

The aquaporin (Aqp) family consists of membrane proteins that are responsible for transporting water and small molecules across biological membranes, and play essential roles in maintaining body fluid homeostasis (Agre et al., 2002; Borgnia et al., 1999; Login & Nejsum, 2023). Seventeen Aqp genes, designated *aqp0*–*aqp16*, have been identified in vertebrates (Chauvigne et al., 2019; Finn et al., 2014; Finn & Cerda, 2015). In this article, the protein names of all species are shown with the first letter capitalized, and the gene names of all species are shown as lowercase and italicized letters. Their gene composition changed significantly over the evolutionary history of each lineage. For instance, *aqp2*, *aqp5*, and *aqp6* exist in tandem on mammalian chromosomes, but are absent in cartilaginous and ray‐finned fish. This indicates that they are members uniquely acquired by the tetrapods. Conversely, teleosts possess numerous *aqp* paralogs owing to tandem duplications in the common ancestor of ray‐finned fish and whole‐genome duplications in the common ancestor of teleosts (Finn et al., 2014; Finn & Cerda, 2015; Yilmaz et al., 2020). Thus, a comparative analysis of gene composition across various lineages is crucial for understanding the evolution of the *aqp* family.

Chondrichthyans are the earliest‐diverging clade of jawed vertebrates and play a crucial role in understanding the ancestral forms of vertebrate genes and their physiological functions. In the context of osmoregulation, cartilaginous fish exhibit characteristics distinct from those of other vertebrates. Many inhabit seawater and employ a strategy of maintaining an osmotic pressure equal to or slightly higher than that of seawater by accumulating high concentrations of urea and trimethylamine *N*‐oxide (TMAO) in their body fluids (Marshall & Grosell, 2006). Consequently, cartilaginous fish can survive in seawater without water loss. Conversely, marine teleost fish actively acquire water by drinking seawater and excreting salts through their gills and kidneys to maintain body fluids at approximately one‐third the osmotic pressure of seawater (Marshall & Grosell, 2006; Takei et al., 2014). Elucidating the composition of *aqp* genes in cartilaginous fish and identifying the specific molecular species involved in urea handling are crucial for understanding their environmental adaptations. Because urea regulation relies on tissue‐specific mechanisms, specifically reabsorption in the kidneys and suppression of leakage in the gills (Part et al., 1998; Wood et al., 2013), research must determine which Aqp isoforms mediate water transport as well as urea retention and excretion.

Extensive research on Aqp in cartilaginous fish has been reported, with a predominant focus on spiny dogfish (*Squalus acanthias*). Cutler et al. revealed the localization of Aqp4 in the kidney using immunohistochemistry (Cutler et al., 2011, 2012). Based on localization analysis of the urea transporter (Ut), a model for urea reabsorption in cartilaginous fish kidneys has been proposed (Hyodo et al., 2014). Furthermore, immunohistochemistry of the spiny dogfish kidney revealed the localization of Aqp3 and Aqp15 in the basolateral and apical membranes of the tubule, respectively (Cutler, Kurt, et al., 2022; Cutler, Murray, et al., 2022). The spiny dogfish (*S. acanthias*) possesses Aqp8, which is not expressed in the gills, kidneys, or digestive tract but is expressed in the brain (Cutler, Mainer, & Ojo, 2022). However, the presence of Aqp8 has not been confirmed in other cartilaginous fish (Finn et al., 2014; Finn & Cerda, 2015; Kuraku et al., 2024). Additionally, evidence suggests that spiny dogfish Aqp11 may be pseudogenized (Cutler et al., 2024), whereas spiny dogfish Aqp12 is expressed in the stomach and liver (Cutler et al., 2025). In recent years, Aqp1 localization in the gills, intestines, rectal glands, and kidneys of spiny dogfish has been revealed by immunohistochemistry (Cutler & Maciver, 2025), along with that of Aqp0 in organs including the kidneys (Cutler et al., 2026). These results contributed to a growing understanding of the overall localization pattern of the Aqp family, particularly within the spiny dogfish kidneys (Cutler, Kurt, et al., 2022).

In 2007, the genome of the elephant shark (*Callorhinchus milii*) was sequenced by Venkatesh et al., marking the first genome sequencing of a cartilaginous fish, the elephant shark (Venkatesh et al., 2007, 2014). Subsequently, in 2018, Hara et al. reported the sequencing of the cloudy catshark (*Scyliorhinus torazame*), brownbanded bamboo shark (*Chiloscyllium punctatum*), and whale shark (*Rhincodon typus*) genomes, and in 2019, Marra reported the genome of the great white shark (*Carcharodon carcharias*) (Hara et al., 2018; Marra et al., 2019). Concurrently, as a component of the Vertebrate Genome Project, the genomes of various cartilaginous fish species, including thorny skate, were subjected to whole‐genome sequencing (Rhie et al., 2021). In the 2020s, the genomes of the white‐spotted bamboo shark (*Chiloscyllium plagiosum*) (Wagner et al., 2024), little skate (*Leucoraja erinacea*) (Marletaz et al., 2023), and spiny dogfish (*S. acanthias*) (Wagner et al., 2023) were investigated. As a component of the Darwin Tree of Life Project, the genomes of various cartilaginous fish species, including the smaller spotted catshark (*Scyliorhinus canicula*), have been subjected to whole‐genome sequencing (Darwin Tree of Life Project, C, 2022). Within the subclass Holocephali, the genomes of the small‐eyed rabbitfish (*Hydrolagus affinis*) and the silver chimera (*Chimera phantasma*) have been sequenced (Fonseca et al., 2020; Teramura et al., 2026). Notably, in addition to those not yet published, the National Center for Biotechnology Information (NCBI) currently lists over 60 cartilaginous fish genome sequences. Advances in genome sequencing have elucidated the structure of the aquaporin (Aqp) family in cartilaginous fish. Some of these resources are fragmented, and the functional properties of the encoded proteins have not been systematically validated. Therefore, a comprehensive analysis is required to bridge the gap between genomic data and physiological function.

In 2014, Finn et al. conducted a comparative genome analysis of various vertebrates to elucidate the overall genetic composition of the *aqp* family in vertebrates (Finn et al., 2014; Finn & Cerda, 2015). Subsequent detailed analyses by Yilmaz et al. demonstrated the presence of chondrichthyan‐specific paralogs of *aqp3* and *aqp10*, naming them *aqp3c1*, *aqp3c2*, *aqp10c1*, and *aqp10c2* (where “c” in c1 and c2 derives from Chondrichthyes) (Yilmaz et al., 2020). Furthermore, although Aqp8 is conserved in many vertebrates, it has not been identified in any cartilaginous fish, except for the spiny dogfish (*S. acanthias*). As a result, the distribution and loss of *aqp8* in cartilaginous fish remain unclear. Furthermore, no comprehensive measurements have been conducted to assess the activity of the *aqp* gene products found in cartilaginous fish in previous studies.

This study aimed to elucidate the gene composition and molecular evolution of the *aqp* gene family in cartilaginous fish and to comprehensively analyze the activities of the proteins they encode, providing a foundation for understanding their physiological roles. To achieve this, we comprehensively searched publicly available genome and protein databases and performed molecular phylogenetic and synteny analyses to confirm the universal *aqp* gene composition in cartilaginous fish. Second, species were selected based on the results of the molecular phylogenetic analysis. Using species such as the sharpnose sevengill shark (*Heptranchias perlo*), the activities of aquaglyceroporins and Aqp8 were experimentally analyzed using *Xenopus* oocyte swelling assays. This study provides foundational information to improve our understanding of the diversity of Aqp functions in cartilaginous fish and their evolutionary backgrounds.

## 2 MATERIALS AND METHODS

### 2.1 Identification of the *aqp* family in cartilaginous fish genome databases

The predicted amino acid sequences and accession numbers encoded by the *aqp* family were obtained from the genome databases of the six cartilaginous fish species listed in Table 1. High‐quality genome data of sharpnose sevengill shark (*Heptranchias perlo*) and North Pacific spiny dogfish (*Squalus suckleyi*) have been decoded by the Vertebrate Genomes Project (GCA_035084215.1) and Canadian BioGenome Project (GCA_026260435.1), respectively. The divergence times of the species were retrieved from the TimeTree database (http://www.timetree.org/) (Kumar et al., 2022). The amino acid sequences of Aqps in human (*Homo sapiens*), mouse (*Mus musculus*), Australian saltwater crocodile (*Crocodylus porosus*), Western clawed frog (*Xenopus tropicalis*), coelacanth (*Latimeria chalumnae*), spotted gar (*Lepisosteus oculatus*), and zebrafish (*Danio rerio*) are listed in Table S1. These sequences served as queries for BLASTP and TBLASTN analyses to identify each Aqp member in each cartilaginous fish species using the National Center for Biotechnology Information (NCBI) BLAST server (BLAST+ versions 2.15.0, 2.16.0, and 2.17.0; https://blast.ncbi.nlm.nih.gov) (Camacho et al., 2009; Johnson et al., 2008) or Ensembl genome browser (versions 112–114; https://www.ensembl.org) (Martin et al., 2023). The amino acid sequences and accession numbers of Aqp members of each cartilaginous fish species were collected (Table 2). The Aqps of the sharpnose sevengill shark and North Pacific spiny dogfish, along with several other Aqps, were not annotated; thus, they were annotated manually (Table S2).

**TABLE 1:** Cartilaginous fish genome databases analyzed in this study.

Species	Genome database	References
Elephant shark (*Callorhinchus milii*)	GCA_018977255.1	(Venkatesh et al., 2007, 2014)
Great white shark (*Carcharodon carcharias*)	GCA_017639515.1	(Wagner et al., 2024)
Whitespotted bambooshark (*Chiloscyllium plagiosum*)	GCA_004010195.1	(Zhang et al., 2020)
Epaulet shark (*Hemiscyllium ocellatum*)	GCA_020745735.1	(Sendell‐Price et al., 2023)
Sharpnose sevengill shark (*Heptranchias perlo*)	GCA_035084215.1	Vertebrate Genomes Project (Rhie et al., 2021)
North Pacific spiny dogfish (*Squalus suckleyi*)	GCA_026260435.1	Canadian BioGenome Project (https://earthbiogenome.ca/)

**TABLE 2:** Accession numbers of Aqps in various cartilaginous fish species.

	Elephant shark (*Callorhinchus milii*)	Great white shark (*Carcharodon carcharias*)	Whitespotted bambooshark (*Chiloscyllium plagiosum*)	Epaulet shark (*Hemiscyllium ocellatum*)	Sharpnose sevengill shark (*Heptranchias perlo*)	North pacific spiny dogfish (*Squalus suckleyi*)
*aqp0*	XP_042200078.1	XP_041036095.1	XP_043537871.1	XP_060677156.1	XP_067832415.1	Table S2
*aqp1*	Table S2	XP_041040070.1	XP_043546996.1	XP_060681184.1	XP_067824374.1	Table S2
*aqp3c1*	XP_007895330.1	Table S2	XP_043541722.1	XP_060692140.1	XP_067840664.1	Table S2
*aqp3c2*	XP_007895329.1	XP_041054146.1	XP_043541616.1	XP_060685563.1	XP_067840639.1	Table S2
*aqp4*	XP_007893283.1	XP_041045693.1	XP_043543564.1	XP_060680519.1	XP_067833995.1	Table S2
*aqp8*					XP_067859851.1	Table S2
*aqp9*	XP_007904652.1	XP_041030986.1	XP_043533387.1	XP_060709247.1	XP_067827607.1	Table S2
*aqp10c1*		XP_041037505.1	Table S2	XP_060676579.1	XP_067832134.1	Table S2
*aqp10c2*	XP_042202609.1 XP_042190669.1	Table S2	Table S2	XP_060676581.1	XP_067832133.1	Table S2
*aqp11*		XP_041055688.1	XP_043548363.1	XP_060682285.1	XP_067841784.1	Table S2
*aqp12*	XP_042195190.1	XP_041061780.1	XP_043558832.1	XP_060689942.1	XP_067850912.1	Table S2
*aqp14*	QKE23205.1	XP_041036094.1	XP_043537870.1	XP_060677007.1	XP_067832416.1	Table S2
*aqp15*	XP_007905580.2	XP_041029214.1	XP_043541015.1	XP_060704562.1	XP_067825249.1	Table S2

### 2.2 Phylogenetic analysis of the Aqp family in cartilaginous fish

The amino acid sequences of Aqps listed in Table 2, Table S1 were aligned using ClustalW software (Chenna et al., 2003) (version 1.8) on the GenomeNet server (https://www.genome.jp/tools‐bin/clustalw). The amino acid sequences of human Aqp‐0f12, Western clawed frog Aqp‐13, −14, and −16, and coelacanth Aqp15 were used for comparison. The evolutionary history was inferred using the Maximum Likelihood method via IQ‐TREE (version 1.6.11) (Trifinopoulos et al., 2016) (https://www.hiv.lanl.gov/content/sequence/IQTREE/iqtree.html). ModelFinder identified the Jones–Taylor–Thornton (JTT) + F + G4 model (Jones et al., 1992) as the best‐fit substitution model based on the lowest Bayesian information criterion (BIC). The tree with the highest log‐likelihood (−28024.157) is presented. The percentages of trees in which the associated taxa were clustered together were calculated using 1000 ultrafast bootstrap approximations (Hoang et al., 2018). The alignment comprised 91 sequences with 669 columns, 485 distinct patterns, 343 parsimony‐informative sites, 111 singleton sites, and 215 constant sites. The resulting Newick format tree was visualized the MEGA12 software (version 12.0.11) (Kumar et al., 2024).

### 2.3 Synteny analysis of *aqp3*, *aqp9*, and *aqp8* in cartilaginous fish

Genome databases of the six cartilaginous fish species listed in Table 1 were analyzed. Synteny of the genomic regions listed in Tables S3–S5 was analyzed using the NCBI Genome Data Viewer (versions 3.49–3.51; https://www.ncbi.nlm.nih.gov/genome/gdv/) (Rangwala et al., 2021) and the Ensembl genome browser (versions 112–114; https://www.ensembl.org) (Martin et al., 2023).

### 2.4 Expression vector construction

A plasmid expressing Aqp in *Xenopus laevis* oocytes was constructed by inserting a cDNA translation frame into the pGEMHE vector (Liman et al., 1992). The full ‐ length cDNA translation regions of the sharpnose sevengill shark (*H. perlo*, Hpe) Aqp3c1, Aqp3c2, Aqp8, Aqp10c1, Aqp10c2; elephant shark (*C. milii*, Cmi) Aqp3c1, Aqp3c2; and whitespotted bambooshark (*Chiloscyllium plagiosum*, Cpl) Aqp3c1 and Aqp3c2 were each artificially extended at the 3′ end with a 15‐base sequence (3′‐GCAGATCAATTCCCC, GGGGATCCGAATTCT‐5′) and synthesized (Eurofins Genomics, Tokyo, Japan). This was then inserted into the pGEMHE vector using NEBuilder HiFi DNA Assembly (New England Biolabs, Ipswich, MA, USA; Cat. No. E2621L). The constructed plasmid vector was purified using the FastGene Plasmid Mini Kit (Nippon Genetics, Tokyo, Japan; Cat. No. FG‐90502), and sequence analysis confirmed the absence of amino acid mutations. The Aqp expression vector was purified using NucleoBond Xtra Midi (Macherey‐Nagel, Düren, Germany; Cat. No. 740410.100), linearized with the NotI restriction enzyme (Takara Bio, Kusatsu, Japan; Cat. No. 1166B), and purified using the Wizard SV Gel and PCR Clean‐Up System (Promega, Madison, WI, USA; Cat. No. A9282). Capped sense RNA (cRNA) was synthesized from the linearized Aqp expression vector using the mMessage mMachine T7 kit (Thermo Fisher Scientific, Waltham, MA, USA; Cat. No. AM1344).

### 2.5 Expression of Aqps in *Xenopus* oocytes and swelling assay

*X. laevis* oocytes were dissociated with collagenase (Sigma‐Aldrich, St. Louis, MO, USA; Cat. No. C9891‐5G), as previously described (Kumagai et al., 2023; Romero, Kanai, et al., 1998; Ushio et al., 2022), and injected with 50 nL of water or a solution containing 0.5 ng/nL cRNA (25 ng/oocyte) using the Nanoject‐II injector (Drummond Scientific, Broomall, PA, USA; Cat. No. 3‐000‐204‐KIT). Oocytes were incubated in OR3 medium at 16°C and observed for 4–5 days after injection. The OR3 medium (1 L) consisted of 0.7% w/v powdered Leibovitz's L‐15 medium with L‐glutamine (Thermo Fisher Scientific; Cat. No. 41300‐070), 50 mL of 10,000 U penicillin, 10,000 U streptomycin solution in 0.9% NaCl (Nacalai Tesque, Kyoto, Japan; Cat. No. 09367‐34), and 5 mM HEPES (pH 7.50) (Romero, Fong, et al., 1998; Romero, Kanai, et al., 1998). Osmolality was adjusted to 200 mOsmol/kg using NaCl powder.

Oocyte swelling was monitored using a stereomicroscope (SZX9; Olympus, Tokyo, Japan) equipped with a charge‐coupled device camera (DS‐Fi2; Nikon, Tokyo, Japan), as previously described (Kumagai et al., 2023; Nagashima et al., 2025; Nagashima & Kato, 2026; Ushio et al., 2022). An in‐focus image of the oocyte was captured every 30 s for a total of 10 min from an overhead perspective. The volume and surface area of the oocyte were calculated based on the measured diameter in each image, assuming spherical geometry. Oocytes incubated with ND96 (approximately 200 mOsmol/kg) were transferred to 2‐fold‐diluted ND96 (approximately 100 mOsmol/kg) for water transport assays. For the glycerol, urea, and boric acid transport assays, oocytes were transferred to an isotonic solution containing ND96 supplemented with 180 mM glycerol, urea, or boric acid instead of NaCl and adjusted to an osmolality of approximately 200 mOsmol/kg. Water permeability (*P*_water_) was calculated from the osmotic swelling data and the molar volume of water (*V*_*w*_ = 18 cm^3^/mol) as follows (Preston et al., 1992): *P*_water_ = [*V*_*o*_ × *d*(*V*/*V*_*o*_)/*dt*]/[*S* × *V*_*w*_ × (osm_in_ – osm_out_)], where *S* is the initial oocyte surface area. The solute permeability (*P*_solute_) was calculated from the swelling data, total osmolality of the system (osm_total_ = 200 mOsmol/kg), and solute gradient (sol_out_ – sol_in_) as follows (Carbrey et al., 2003): *P*_solute_ = osm_total_ × [*V*_*o*_ × *d*(*V*/*V*_*o*_)/*dt*]/[*S* × (sol_out_ – sol_in_)]. The water, glycerol, urea, and boric acid transport activities of each aquaglyceroporins and Aqp8 were evaluated using oocytes from the same animal, and the experiment was repeated using at least three frogs. *P*_*water*_ and *P*_*solute*_ values were compared between Aqp‐expressing and control oocytes, and statistical significance was evaluated by Kruskal–Wallis non‐parametric test, followed by Dunn's multiple comparisons test for all pairwise comparisons using GraphPad Prism software (version 8; GraphPad, San Diego, CA, USA).

### 2.6 Ethics statement

All *Xenopus* protocols and procedures were conducted in accordance with the National Institutes of Health Guide for the Care and Use of Laboratory Animals and were approved by the Institutional Animal Experiment Committee of the Institute of Science, Tokyo. This study did not involve human participants, and therefore informed consent was not required.

## 3 RESULTS

### 3.1 Composition of *aqp* in cartilaginous fishes and relationship to those in other jawed vertebrates

**FIGURE 1:**
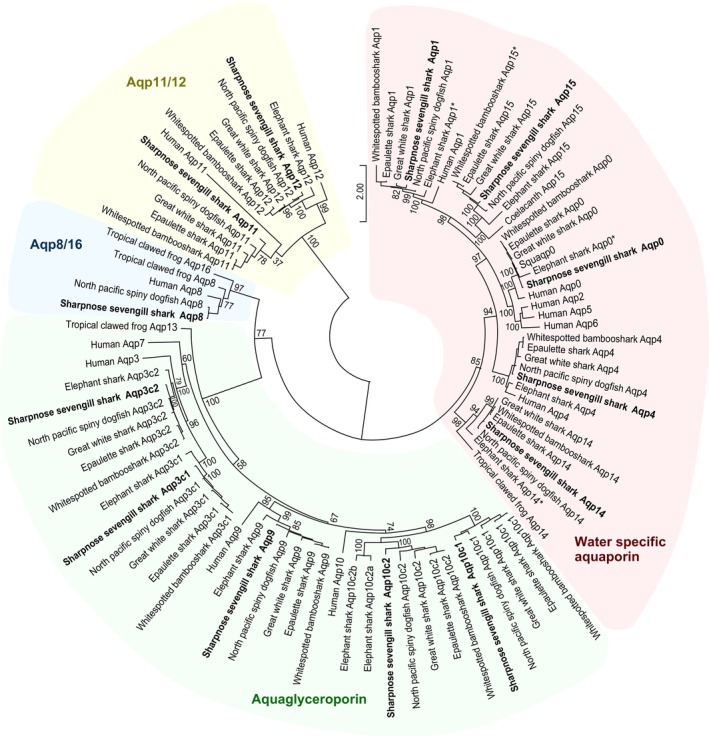
Phylogenetic relationship of Aqps in cartilaginous fish. A phylogenetic tree based on the amino acid sequences of Aqps in 12 cartilaginous fish species is shown. The phylogenetic tree was generated using the maximum‐likelihood method under the JTT + F + G4 model. The amino acid sequences used for the phylogenetic tree are shown in Table 2, Table S2. The numbers indicate bootstrap values. Asterisk (*) indicates sequences for which only partial data were found.

To elucidate the genetic composition and overall molecular evolution of the cartilaginous fish *aqp* family, we comprehensively identified *aqp* genes across the entire genome, using genomic data from five species of the Elasmobranchii subclass and one species within the Holocephali subclass (Table 1). We obtained the predicted amino acid sequences and accession numbers of these genes (Table 2, Table S1). Molecular phylogenetic analysis revealed that each Aqp present in cartilaginous fish formed a clade with orthologs from other vertebrates (Figure 1). As previously reported by Yilmaz et al. (Yilmaz et al., 2020), the composition was *aqp0, 1, 3c1, 3c2, 4, 8, 9, 10c1, 10c2, 11, 12, 14, 15*, revealing that cartilaginous fish retain 13 distinct *aqp* genes.

### 3.2 Synteny analysis of *aqp3*, *aqp9*, and *aqp8* in cartilaginous fish

**FIGURE 2:**
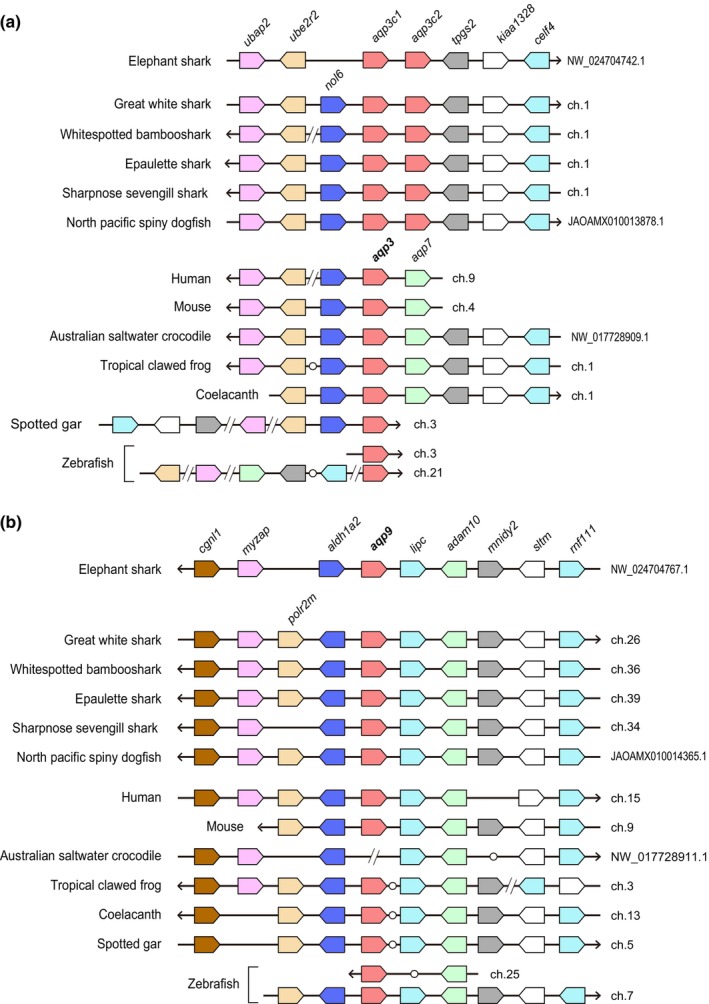
Synteny analyses of *aqp3* and *aqp9* genes in various cartilaginous fish species. The figure shows the synteny around *aqp3* and *aqp9* in various cartilaginous fish. Boxes with arrows indicate each gene and its transcription direction, while the arrow orientation represents the genome orientation defined in the database. Open circles indicate the presence of one gene between the two genes.

**FIGURE 3:**
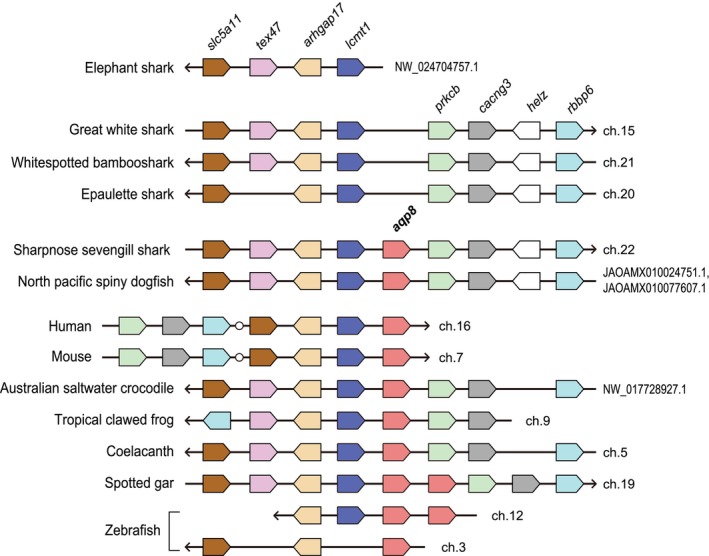
Synteny analyses of *aqp8* in various cartilaginous fish species. The figure shows the synteny around *aqp8* in various cartilaginous fish. Boxes with arrows indicate each gene and its transcription direction, and the arrow orientation represents the genome orientation defined in the database. Open circles indicate the presence of one gene between the two genes.

The synteny of *aqp10* is conserved in cartilaginous fish and other vertebrates (Nagashima & Kato, 2026). This study examined the synteny of *aqp3c1, 3c2, 8*, and *9* in cartilaginous fish (Figures 2 and 3). The results indicated that the synteny surrounding *aqp3c1, 3c2, 8*, and *9* in cartilaginous fish is highly conserved. Clear similarities were also observed among representative vertebrate groups, including mammals, reptiles, amphibians, lobe‐finned fish, and bony fish.

### 3.3 Composition of *aqp* genes in cartilaginous fish

**FIGURE 4:**
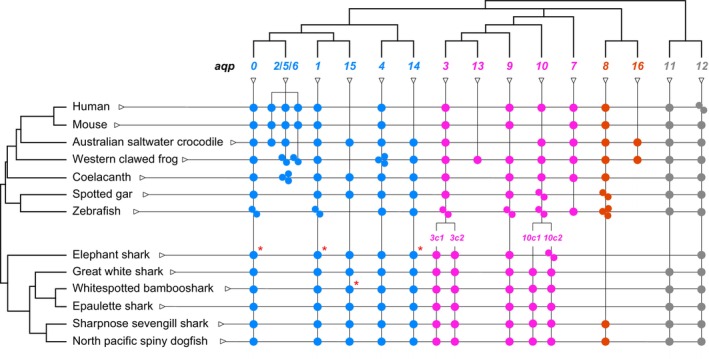
Composition of *aqp*s in cartilaginous fishes and comparison with tetrapods and ray‐finned fishes. Each dot represents an *aqp* gene and its copy number. Animal species and accession numbers are listed in Table 2, Table S2. Species divergence times were retrieved from the TimeTree database (http://www.timetree.org/) (Kumar et al., 2022) and are shown on the left. Asterisk (*) indicates sequences for which only partial data were found.

Figure 4 summarizes the *aqp* genes present in the cartilaginous fish. The gene composition of water‐specific and non‐orthodox Aqps is well conserved across species. All cartilaginous fish species analyzed in this study possessed *aqp3c1* and *aqp3c2* as two tandem paralogs of *aqp3*. The elephant shark (*C. milii*), which belongs to the Holocephali subclass, possesses two copies of *aqp10c2* (Nagashima & Kato, 2026; Yilmaz et al., 2020). In many members of the subclass Elasmobranchii, both tandemly arranged *aqp10c1* and *aqp10c2* were retained. *aqp8* was detected in only two species: the sharpnose sevengill shark (*H. perlo*) and North Pacific spiny dogfish (*S. suckleyi*). These two species were confirmed to possess all *aqp* genes found in cartilaginous fish.

### 3.4 Water and solute permeabilities of the sharpnose sevengill shark aquaglyceroporins and Aqp8 expressed in *Xenopus* oocytes

**FIGURE 5:**
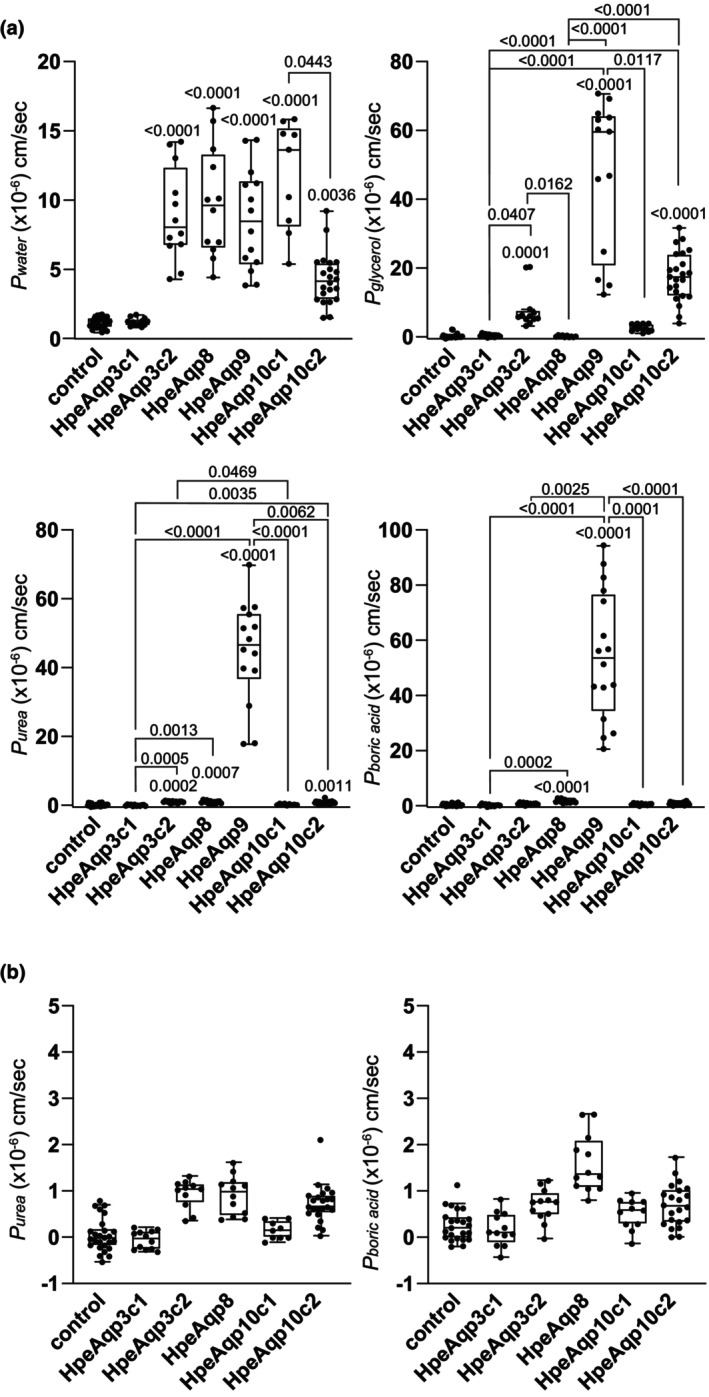
Water and solute permeabilities of aquaglyceroporins and Aqp8 in sharpnose sevengill shark. Aquaglyceroporins and Aqp8 were expressed in *Xenopus* oocytes, and their activity was measured using a swelling assay with hypotonic solutions or isotonic solutions containing glycerol, urea, or boric acid. The permeability [×10^−6^ cm/s] for each species is shown in box‐and‐whisker plots, using the water permeability coefficient (*P*_water_) calculated under hypotonic conditions and the solute permeability coefficient (*P*_solute_) calculated in the presence of solutes as indices. Water‐injected oocytes were utilized as the negative control. The Kruskal–Wallis test was employed for intergroup comparisons, and Dunn's multiple comparison test was utilized for post hoc analysis. Significant *p*‐values are provided numerically, with values for comparison against the control positioned directly above the respective boxes. Other significant pairwise *p*‐values (*p* < 0.05) are indicated above solid lines connecting the compared groups. All *p*‐values are shown in Table S6. Data are presented as interquartile range (25th–75th percentiles) (box), whiskers representing the range, outliers (>1.5× the interquartile range above the upper quartile), and median (line in the box). All individual data points are shown.

*X. laevis* oocytes expressing *H. perlo* (Hpe) Aqp3c1, 3c2, 8, 9, 10c1, and 10c2 were analyzed for water, glycerol, urea, and boric acid permeability using swelling assays (Figure 5, Table 3). Oocytes expressing all the analyzed Aqps, except Aqp3c1, showed a significant increase in water permeability (*P*_*water*_) under hypotonic conditions. In an isotonic solution containing 180 mM glycerol, a significant increase in glycerol permeability (*P*_*glycerol*_) was observed in oocytes expressing HpeAqp3c2, 9, or 10c2. In an isotonic solution containing 180 mM urea, oocytes expressing HpeAqp3c2, 8, 9, and 10c2 showed a significant increase in urea permeability (*P*_*urea*_). HpeAqp9 exhibited particularly strong permeability (*P*_*urea*_ mean >40 × 10^−6^ cm/s), whereas HpeAqp3c2, 8, 10c2 showed weak permeability (*P*_*urea*_ mean <5 × 10^−6^ cm/s). In an isotonic solution containing 180 mM boric acid, HpeAqp8 and HpeAqp9 significantly increased the boric acid permeability (*P*_boric acid_) in oocytes. HpeAqp9 showed strong permeability (*P*_boric acid_ mean >40 × 10^−6^ cm/s), whereas HpeAqp8 showed weak permeability (*P*_boric acid_ mean <5 × 10^−6^ cm/s).

**TABLE 3:** Water and solute permeabilities of sharpnose sevengill shark Aqps.

Protein	*P*_water_ (×10^−6^ cm/s, 100 mosM inside osmotic gradient)	*P*_glycerol_ (×10^−6^ cm/s, 180 mM outside solute gradient)	*P*_urea_ (×10^−6^ cm/s, 180 mM outside solute gradient)	*P*_boric acid_ (×10^−6^ cm/s, 180 mM outside solute gradient)
Control	1.1 ± 0.4 (26)	1.8 ± 0.6 (27)	0.1 ± 0.4 (26)	0.3 ± 0.3 (23)
HpeAqp3c1	1.2 ± 0.3 (12)	0.3 ± 0.4 (12)	−0.1 ± 0.2 (12)	0.3 ± 0.3 (12)
HpeAqp3c2	8.9 ± 3.3 (12)	8.0 ± 5.6 (12)	0.9 ± 0.3 (12)	0.7 ± 0.3 (12)
HpeAqp8	9.9 ± 3.8 (12)	0.1 ± 0.2 (10)	0.9 ± 0.4 (12)	1.6 ± 0.6 (12)
HpeAqp9	8.6 ± 3.5 (14)	47.1 ± 21.3 (13)	44.5 ± 14.4 (14)	54.6 ± 22.7 (16)
HpeAqp10c1	11.8 ± 3.7 (9)	2.6 ± 1.0 (12)	0.2 ± 0.2 (9)	0.5 ± 0.3 (11)
HpeAqp10c2	4.3 ± 1.8 (22)	17.6 ± 7.0 (23)	0.7 ± 0.4 (23)	0.7 ± 0.4 (22)
CmiAqp3c1	1.4 ± 0.5 (12)	0.1 ± 0.2 (10)	−0.2 ± 0.3 (11)	0.1 ± 0.3 (11)
CmiAqp3c2	10.8 ± 5.0 (12)	21.9 ± 7.0 (10)	1.5 ± 0.8 (9)	0.8 ± 0.4 (12)
CplAqp3c1	1.2 ± 0.3 (11)	−0.1 ± 0.3 (10)	−0.1 ± 0.3 (11)	0 ± 0.3 (12)
CplAqp3c2	10.7 ± 3.0 (12)	4.3 ± 1.9 (12)	0.5 ± 0.3 (12)	0.5 ± 0.4 (12)

*Note*: Quantitative data are presented as the mean ± standard deviation. Numbers in parentheses indicate the total number of oocytes analyzed.

### 3.5 Water and solute permeabilities of the elephant shark and whitespotted bambooshark Aqp3s expressed in *Xenopus* oocytes

**FIGURE 6:**
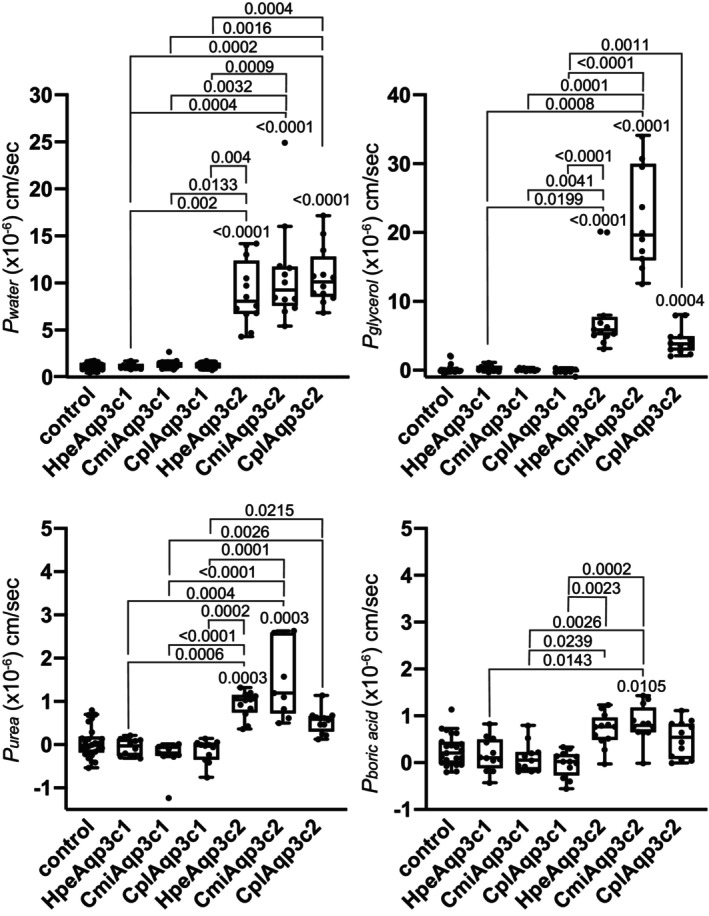
Activity of Aqp3c1 and 3c2 from the elephant shark and whitespotted bambooshark. Aqp3c1 and 3c2 from elephant sharks and whitespotted bambooshark were expressed in *Xenopus* oocytes, and their activity was measured using swelling assays with hypotonic solutions (0.5× ND96) or isotonic solute solutions (glycerol, urea, boric acid). The permeability [×10^−6^ cm/s] for each species is shown in box‐and‐whisker plots, using the water permeability coefficient (*P*_*water*_) calculated under hypotonic conditions and the solute permeability coefficient (*P*_*solute*_) calculated in the presence of solutes as indices. Oocytes injected with nuclease‐free water served as the negative control. The Aqp proteins analyzed were CmiAqp 3c1 and 3c2 from the elephant shark and CplAqp 3c1 and 3c2 from the whitespotted bambooshark. The Kruskal–Wallis test was used for intergroup comparisons, and Dunn's multiple comparison test for post hoc analysis. The significance level was set at less than 5% (*p* < 0.05), and groups labeled with different letters indicate statistically significant differences. Significant *p*‐values are provided numerically. Values for comparison against the control are positioned directly above the respective boxes. Other significant pairwise *p*‐values (*p* < 0.05) are indicated above the solid lines connecting the compared groups. All *p*‐values are presented in Table S7. Values are presented as interquartile range (25th–75th percentiles) (box), whiskers representing the range, outliers (>1.5× the interquartile range above the upper quartile), and median (line in the box). All individual data points are shown.

Because water transport activity could not be obtained for HpeAqp3c1, we additionally analyzed the transport activities of Aqp3c1 and 3c2 from the elephant shark (*C. milii*, Cmi) and the whitespotted bambooshark (*C. plagiosum*, Cpl). Under hypotonic conditions, oocytes expressing Aqp3c2 exhibited a significant increase in water permeability (*P*_*water*_) (Figure 6, Table 3). In contrast, no increase in water permeability was observed in oocytes expressing Aqp3c1. Oocytes expressing Aqp3c2 exhibited significantly increased glycerol permeability (*P*_glycerol_) (Figure 6).

## 4 DISCUSSION

Molecular phylogenetic analysis confirmed that 13 members of *aqp* genes are conserved across cartilaginous fish. This result is consistent with previous reports by Finn et al. (Finn et al., 2014; Finn & Cerda, 2015; Yilmaz et al., 2020) and (Kuraku et al. (2024)). Notably, *aqp8*, which has previously been reported only in the spiny dogfish (*S. acanthias*) (Cutler, Mainer, & Ojo, 2022), was also confirmed in the present study in the sharpnose sevengill shark (*H. perlo*) and North Pacific spiny dogfish (*S. suckleyi*), but not in the other examined species. The sharpnose sevengill shark belongs to the order Hexanchiformes, whereas the spiny dogfish and North Pacific spiny dogfish belong to the order Squaliformes. Both are cartilaginous fish that are evolutionarily closely related (Tanaka et al., 2013). This suggests that although the common ancestor of cartilaginous fishes possessed *aqp8*, a secondary loss occurred in many lineages, excluding those of Hexanchiformes and Squaliformes, during evolution. Regarding *aqp10*, it was inferred that a tandem duplication in the common cartilaginous fish ancestor generated two paralogs, *aqp10c1* and *aqp10c2*, which have evolved independently in different lineages. In elephant sharks, *aqp10c1* was lost, whereas *aqp10c2* was duplicated. The two species, the sharpnose sevengill shark (*H. perlo*) and the North Pacific spiny dogfish (*S. suckleyi*), retained all 13 *aqp* genes present in cartilaginous fish, with no deletions. These species were identified as reference species to examine the molecular evolution of the cartilaginous fish *aqp* family.

Activity measurements via a swelling assay showed that HpeAqp3c2, 9, and 10c2 exhibited glycerol permeability, indicating that these molecules retained the conserved function of aquaglyceroporins. Among the chondrichthyan‐specific Aqp10 paralogs, HpeAqp10c2 exhibited glycerol permeability, whereas no glycerol permeability was detected for HpeAqp10c1. This is thought to result from functional differentiation between Aqp10 paralogs after gene duplication. The activity of HpeAqp10c1 resembled that of water‐specific Aqps rather than that of aquaglyceroporins. A similar functional difference between Aqp10c1 and c2 has been reported in other cartilaginous fish by (Nagashima & Kato, 2026), suggesting that this functional differentiation originated from the ancestral species of cartilaginous fish.

HpeAqp9 exhibited broad solute specificity, with high permeability to all tested solutes, including water, glycerol, boric acid, and, most notably, urea. The fact that HpeAqp9 alone showed a particularly strong urea transport capacity supports the possibility that this molecule is deeply involved in the movement and retention of urea across the cell membrane, contributing to the physiological strategy of cartilaginous fish to accumulate high concentrations of urea in their body fluids. In contrast, no transport activity for water, glycerol, urea, or boric acid was detected in Aqp3c1 derived from three cartilaginous fish species: the sharpnose seven‐gill shark, white‐spotted dogfish, and elephant shark. Further analysis is needed to determine why this experimental system did not detect activity. The following possibilities are under consideration. First, Aqp3c1 normally functions at the cell membrane, but in the *Xenopus* oocyte expression system, it may have failed to translocate to the membrane for some reason, preventing detection of activity in the swelling assay. Second, Aqp3c1 likely normally functions on the cell membrane and is expressed on the membrane in *Xenopus* oocytes. However, its activity requires intracellular signaling to gate it, which cannot be reproduced under the experimental conditions used in oocytes. Third, it is possible that the native functional site of Aqp3c1 is not the cell membrane, but rather organelles. Because Aqp3c1 was not expressed on the cell membrane of *Xenopus* oocytes, it could not be detected with swelling assays. According to immunohistochemical results in spiny dogfish kidneys using anti‐Aqp3c1 antibody by Cutler, Kurt, et al. (2022), Aqp3c1 localizes to the basolateral membrane of tubules. This result supports the first or second possibility above.

The urea permeability of Aqp8 (HpeAqp8) from the cartilaginous fish sharpnose sevengill shark was 0.9 ± 0.4 [×10^−6^ cm/s], indicating relatively low activity. The urea transport capacity of Aqp8 varies significantly across lineages. In ray‐finned fish, Aqp8bb from the Japanese pufferfish (*Takifugu rubripes*) exhibits extremely high permeability at 20.8 ± 12.2 × 10^−6^ cm/s (Kumagai et al., 2023), functioning as a potent urea channel. In contrast, mammalian human Aqp8 has a urea permeability of 0.5 ± 0.5 × 10^−6^ cm/s (Kumagai et al., 2023; Ushio et al., 2022), indicating low activity. Comparing the HpeAqp8 values obtained in this experiment with data from previous studies, the permeability observed was not as high as that of pufferfish Aqp8bb; rather, it was closer to the activity value of human Aqp8. This suggests that Aqp8 exists in both high‐ and low‐urea transport activity types. Furthermore, future studies comparing the structure and activity of Aqp8 in ray‐finned fish, mammals, and cartilaginous fish could potentially analyze the mechanisms that limit urea permeability. Presently, it remains unclear which activity represents the ancestral form. It is expected that analysis of the activity of diverse Aqp8 paralogs, including those in ray‐finned fish, will elucidate the history of the diversification of Aqp8 function.

## AUTHOR CONTRIBUTIONS

**Shinichiro Hidaka:** Conceptualization; formal analysis; investigation; project administration; visualization. **Ayumi Nagashima:** Formal analysis; funding acquisition; investigation; resources; validation; visualization. **Akira Kato:** Conceptualization; data curation; formal analysis; funding acquisition; investigation; methodology; project administration; resources; supervision; validation; visualization.

## FUNDING INFORMATION

This study was supported by the Japan Society for the Promotion of Science (JSPS) KAKENHI, grant numbers 21H02281 (to A.K.) and 21K14781 (to A.N.); a Shiseido Female Researcher Science Grant (to A.N.), which was used to support research activities; the Institute of Science Tokyo Challenging Research Award (to A.N.); and the Temporary Assistant Program by the Support for Work‐Life Balance, DEI Section, Office of Communications, and DEI, Institute of Science Tokyo (to A.N.).

## CONFLICT OF INTEREST STATEMENT

The authors declare no conflict of interest.

## Supporting information


**Table S1.** Accession numbers of Aqps in tetrapods and ray‐finned fishes analyzed in this study.
**Table S2.** Predicted nucleotide and amino acid sequences for cartilaginous fish Aqps.
**Table S3.** Synteny of *aqp3* in the cartilaginous genome databases.
**Table S4.** Synteny of *aqp9* in the cartilaginous genome databases.
**Table S5.** Synteny of *aqp8* in the cartilaginous genome databases.
**Table S6.** *p*‐values from Dunn’s multiple‐comparisons test for Figure 5.
**Table S7.** *p*‐values from Dunn’s multiple‐comparisons test for Figure 6.

## Data Availability

Data will be made available upon request.
