# Supplementary material for: Genome‐wide identification and solute selectivity of aquaporins in the sharpnose sevengill shark, Heptranchias perlo
Source: Physiol Rep. 2026 Apr 24;14(8):e70895. doi: 10.14814/phy2.70895 (PMC13109646; doi:10.14814/phy2.70895)
Supplement: Supplementary file 1 — Table S1. Accession numbers of Aqps in tetrapods and ray‐finned fishes analyzed in this study. Table S2. Predicted nucleotide and amino acid sequences for cartilaginous fish Aqps. Table S3. Synteny of aqp3 in the cartilaginous genome databases. Table S4. Synteny of aqp9 in the cartilaginous genome databases. Table S5. Synteny of aqp8 in the cartilaginous genome databases. Table S6. p‐values from Dunn’s multiple‐comparisons test for Figure 5. Table S7. p‐values from Dunn’s multiple‐comparisons test for Figure 6. [file PHY2-14-e70895-s001.docx]

| **Table S1.** Accession numbers of Aqps in tetrapods and ray-finned fishes analyzed in this study. | | | | | | |  |
| --- | --- | --- | --- | --- | --- | --- | --- |
|  | Human (*Homo sapiens*) | Mouse (*Mus musculus*) | Australian saltwater crocodile (*Crocodylus porosus*) | Toropical clawed frog (*Xenopus tropicalis*) | Coelacanth (*Latimeria chalumnae*) | Spotted gar (*Lepisosteus oculatus*) | Zebra fish (*Danio rerio*) |
| *aqp0* | NP_036196.1 | NP_032626.2 | XP_019409767.1 | NP_001090816.1 | XP_005986314.1 | XP_006629345.1 | NP_001003534.1 NP_001018356.1 |
| *aqp1* | NP_001316801.1 | NP_031498.1 | XP_019384654.1 | NP_001005829.1 | XP_064410541.1 | XP_006634313.1 | NP_996942.1 NP_001129154.1 |
| *aqp2* | NP_000477.1 | NP_033829.3 | XP_019412024.1 |  | XP_005986219.1 XP_064408586.1 XP_005986375.1 |  |  |
| *aqp3* | NP_001305073.1 | NP_057898.2 | XP_019387387.1 | NP_001016845.1 | XP_006004793.1 | XP_006626763.1 | NP_001159593.1 NP_998633.1 |
| *aqp4* | NP_001304313.1 | NP_001295571.1 | XP_019402832.1 | NP_001304774.1 XP_017950427.1 NP_001135583.1 | XP_064409699.1 | XP_006634102.2 | NP_001345242.1 |
| *aqp5* | NP_001642.1 | NP_033831.1 | XP_019412025.1 | NP_001297041.1 NP_001015749.1 |  |  |  |
| *aqp6* | NP_001643.2 | XP_006520413.1 |  | XP_002935778.1 XP_012813770.2 |  |  |  |
| *aqp7* | NP_001161.1 | NP_031499.1 | XP_019387385.1 | NP_001015726.1 | XP_064409331.1 |  | NP_956204.2 |
| *aqp8* | NP_001160.2 | NP_031500.1 | XP_019397382.1 | NP_001107728.1 | XP_005994818.1 | XP_006637203.1 XP_006637204.1 | NP_001073651.1 NP_001004661.1 NP_001108382.2 |
| *aqp9* | NP_001307564.1 | XP_006511395.1 |  | XP_002937719.2 | XP_005998794.1 | XP_015198748.1 | NP_001028268.1 NP_001171215.1 |
| *aqp10* | NP_536354.2 |  | XP_019412394.1 | XP_017945847.1 | XP_005996556.2 | XP_069041222.1  XP_015224218.2 | NP_001002349.1 XP_005159449.1 |
| *aqp11* | NP_001350406.1 | NP_780314.1 | XP_019385984.1 | XP_004912256.2 | XP_006006366.1 | XP_015196614.1 | NP_001314822.1 |
| *aqp12* | NP_945349.1 NP_001095937.1 | NP_808255.1 | XP_019398599.1 | XP_031758818.1 | XP_064419086.1 | XP_006637810.1 | NP_001039327.1 |
| *aqp13* |  |  |  | XP_002940511.2 |  |  |  |
| *aqp14* |  |  | XP_019409780.1 | XP_031752084.1 | QKE23003.1 | XP_015199548.1 | XP_005174182.1 |
| *aqp15* |  |  | XP_019404069.1 |  | XP_064421728.1 | XP_006638402.2 |  |
| *aqp16* |  |  | XP_019398288.1 | XP_002937066.2 |  |  |  |

**Table S2.** Manually predicted nucleotide and amino acid sequences for cartilaginous fish Aqps.

| No. | sequence | size |
| --- | --- | --- |
| 1 | Elephant shark (*Callorhinchus milii*) Aqp1 (partial) |  |
|  | GGCGTCACGCCGGGGCAGGGTGTAGGGGTCGAGCTAATCGTCACCCTCCAGCTGGTTCTCTGCGTCTTCGCCACCACCGACAAGCGCAGGTCCGACCTCTCCGGCTCGGGACCTCTGGCCATCGGGATCTCCGTGGTGATCGGACACCTCCTCGCTATCGGCTTCACGGGCTGCGGGATGAACCCAGCCAGGTCTTTTGGCCCAGCCGTGGTTACCGGACTATTTCAGAATCACTGG | 237 |
|  | GVTPGQGVGVELIVTLQLVLCVFATTDKRRSDLSGSGPLAIGISVVIGHLLAIGFTGCGMNPARSFGPAVVTGLFQNHW | 79 |
| 2 | Great white shark (*Carcharodon carcharias*) Aqp3c1 |  |
|  | ATGACGACTATACTGAAGGTCCAAAGTCTTCTGATGAAACAATGCCTTGCTGAATGTTTAGGAACTTTAATTCATACCATGCTTAGCTGTGGAGTGATAGCACAATTTGTTCTCAGTTATGGTACGCACAAAGAGTTTTTGACTGTTACTTTTGCCAGTGGATTTGCAGTAGCTCTGGGTATATTGGTAACGAGTAAAGTCTCAGGAGCTCACCTGAATCCTGCAGTGACCTTTGCTTTCTGCTTACTTGCTTGTGAACCCTGGTTAAAATTTCCCTTCTTCTTTTTGGCACAAACATTAGGTGCCTTTCTTGGATCAGGAATAATGTTTGGTTTGTATTATGATAAATTGTGGCATTATGGTAATAAACAGCTAACAGTAATTGGAGCAAACTCTACTGCTGGAATATTTACTACTTATCCACATGAACATTTGAATGTAGTAAATGGCATTTTTGATCAGGCAATTGGGACTGCAGCGCTTATACTTTGTATCCTTATCATTGTGGACCCATTGAACAACTCAGTGCCAACAGGACTGGAAGCTTTTACCATTGGCTTTGTGGTTCTGATAATTGGCTGGTCAATGGGTTCAAATTCCCAGTACTCATTAAATCCTGCCAGAGATATTGGACCTCGCTTGTTTACTGCAATTGCTGGTTGGGGATCTGAAGTTTTCAGTGCTGGCAGCTATTGGTTTTGGATCCCCGTTGCCAGCCCAATCATTGGTGCCATATTTGGTGTCCTGATGTATCAGTTCATTGTTGGATTGCGAACTGAGGCAAGAACCAACTGCTCACCCAATGCAGAAGAAAATGTAAAGTTAATGAGCCAGAAACCAAAGGGGAGGTGCTGA | 855 |
|  | MTTILKVQSLLMKQCLAECLGTLIHTMLSCGVIAQFVLSYGTHKEFLTVTFASGFAVALGILVTSKVSGAHLNPAVTFAFCLLACEPWLKFPFFFLAQTLGAFLGSGIMFGLYYDKLWHYGNKQLTVIGANSTAGIFTTYPHEHLNVVNGIFDQAIGTAALILCILIIVDPLNNSVPTGLEAFTIGFVVLIIGWSMGSNSQYSLNPARDIGPRLFTAIAGWGSEVFSAGSYWFWIPVASPIIGAIFGVLMYQFIVGLRTEARTNCSPNAEENVKLMSQKPKGRC | 284 |
| 3 | Great white shark (*Carcharodon carcharias*) Aqp10c2 |  |
|  | ATGAAGAGACTTCGGAAGAAACTCCGGATTAAGAACAAGCTCATTCGGGAATGCCTGGCAGAATTCTTCGGCGTTTACCTGCTGATTTTGATGGGCTCGGCGTCAGTGGCACAGGTTGTATTGTTTTTCGACCGGAAGGGCGAGTACCTGTCAATTGCCTTTGGATACGCTTGCGGGGTCCTGTTCGGCATCTATGCATCAAGAGGAATCTCAGGTGCTCATTTGAACCCCGCCGTCACCTTCAGTCTGTGCCTGCTGGGCCGGTGCCCATGGAAGAAGCTGCCTTTTTACACCATCGCTGAGTGCCTGGGCTCCTTCACTGCCGCAGCGACCACCTTCTGCCTTTATTACGATGCCATATTCGAATTTTCTTACGGGAATCTCACGGTCTGTGGCCCGAGGGCAACAGCGGGAATCTTCGCCACCTACCCCGTCGAGTACCTCAGCCTCCGCAACGGGTTCCTCACCGAGATCATTGGTACCGCTGTTTTGTTAATCTGCATCCTGTGCGTTGGGGATGCCAAAAATGCTGGGGCTCCGGCATTCTTACAACCCCCTCTTGTCGCCACCTCAGTGTTTATCATTGGCATTTGCATGGGTGCCAACACTGGTTACGCCATCAACCCAGCAAGGGACTTCGGACCTCGCTTGTTTACCTATGTAGCTGGGTGGGGCACTGAGGTGTTCACGGCCGGCAACAATTGGTGGTGGATCCCGATCGTCGCCCCGGTCCTGGGGGGGAGCGCGCTGGGCAGCCTGGCCTACGTTCTCCTCATCGAGATGCACCACGAGGACCCCAAGCCACTCAAAGATGTCAAGGACGAGGCAGTCGATGACGCGTAG | 843 |
|  | MKRLRKKLRIKNKLIRECLAEFFGVYLLILMGSASVAQVVLFFDRKGEYLSIAFGYACGVLFGIYASRGISGAHLNPAVTFSLCLLGRCPWKKLPFYTIAECLGSFTAAATTFCLYYDAIFEFSYGNLTVCGPRATAGIFATYPVEYLSLRNGFLTEIIGTAVLLICILCVGDAKNAGAPAFLQPPLVATSVFIIGICMGANTGYAINPARDFGPRLFTYVAGWGTEVFTAGNNWWWIPIVAPVLGGSALGSLAYVLLIEMHHEDPKPLKDVKDEAVDDA | 280 |
| 4 | Whitespotted bambooshark (*Chiloscyllium plagiosum*) Aqp10c1 |  |
|  | ATGGGGAGAGCAGCCACAATCCTGGTCAAAGTCCACGATGCATTTCGATTGAAGAACAAACTCTTCAGAGAGTGTCTGGCAGAGTTCCTGGGGGTCTGCATGTTGATTCTGTTTGGATGTGGAGCTGTAGCGCAGATGGTCGTCAGTAACACGACACGCGGTGAATTCCTGTCGGTCAATCTCGGCTTCGGACTCGGGGCAACGTTCGGAATTTACATCTCCGGAGGGATCTCAGGGGGCCATCTGAATCCGGCCGTGTCCTTCAGTCTGTGCCTGCTTGGCCGGTTCCAATGGAAGAAGTTGCCCTTTTACATGTTCTTCCAGACCCTGGGGGGGTTTGTCGGAGCGGCTGTAGTCTATGGGGTGCATCACGATGGGATCCATGCTGTGAACAACGGGACTTTGTCTGTCACCGGGCCACGTGCCACCGCCTTCATTTTTGGCACCTACCCTGCACCGTTCCTCACCCTTCCCAACGGCGGCTTTATAGACCAGTTGATTGGCACCGGCACCCTCCTCCTGTGCATCTTCGCGGTGGTGGACTCCCAGAACTACGGCGCTCCCAAGATCCTTCAGCCCATCTTCATCGGCCTGTCGGTGGTGGGCATCGGGATGTCCATGGGCTCCAATTCCGGTTATGCCATCAATCCCGCCCGGGACTTCGGGCCGCGTCTGCTCACCCTGGCTGCGGGATGGGGCACCGAGGTCTTCACGGCCGGGAACGGTTGGTGGTGGATCCCCATTGTGGCGCCCATGGTGGGCGCGGTGCTGGGGGCCCTGGTCTACGAGTTGCTGGTGGAGCTGCACCACCTGGAGGCCAAGTCGGGTGCCGCCAACTACGCCGACGAGGAGACGCTGAAGGGGACGGGTGCCGGGGGCAAGGACGGCCCGGCCGTGCGGAGGGACAAGGCCGGAGCAGATGACCAGTTCGTCATGGCGATGTGA | 945 |
|  | MGRAATILVKVHDAFRLKNKLFRECLAEFLGVCMLILFGCGAVAQMVVSNTTRGEFLSVNLGFGLGATFGIYISGGISGGHLNPAVSFSLCLLGRFQWKKLPFYMFFQTLGGFVGAAVVYGVHHDGIHAVNNGTLSVTGPRATAFIFGTYPAPFLTLPNGGFIDQLIGTGTLLLCIFAVVDSQNYGAPKILQPIFIGLSVVGIGMSMGSNSGYAINPARDFGPRLLTLAAGWGTEVFTAGNGWWWIPIVAPMVGAVLGALVYELLVELHHLEAKSGAANYADEETLKGTGAGGKDGPAVRRDKAGADDQFVMAM | 314 |
| 5 | Whitespotted bambooshark (*Chiloscyllium plagiosum*) Aqp10c2 |  |
|  | ATGAGGAGTCTCCGTCAGAAGCTGCAGATCAGGAACAGGCTGGCCCGGGAATGCCTGGCCGAATTCCTCGGGGAATACATGCTCATTCTCATGGGCACAGCGGCAGTGGCTCAGGTGGTGACAAACTTCGATCAGAAAGGGACCTATTTATCGATTAACATTGGCTATGCTGCTGGAGTTCTGTTTGGGATCTACGCCTCAGTTGGAGTCTCAGGGGCTCACCTGAACCCGGCGGTGACCTTCAGCCTGTGCGTCCTGGGCCGGTTTCCCTGGAGAAAGCTTCCGTTCTACACCATCTCCGAGTGCCTGGGGTCATTCGTCGCCTCGGCAACCACGTTCACCCTCTACTACGACGCCATCCACGAGTTCTCTGGTGGCAATCTGACCGTGCGTGGGCCGAGGGGAACAGCTGGCATCTTTGCGACGTATCCCGTCGAGTACCTGTCTGTCCGCAACGGCTTCATAACTGAGGTAATTGGCACCGCTGTTTTGTTGATTTGCGTCTTGAGTGTCGGCGACGCCAAAAATGCTGGTGCCCCGGCTTTCCTCCAGCCGTTGCTGATCTCGGTCTCGGTACTCGTCATCGGTGCTGCTATGGGTGCCAACACCGGCTATGCCATCAACCCAGCGAGAGACCTTGGACCCAGACTCTTCACATTCGTGGCTGGCTGGGGGACTGAAGTTTTCAAAGCCGGGAACGGCTGGTGGTGGATCCCTATCGTCGCGCCCCTGATTGGCGGCGTAATAGGCAGCCTGGCCTACACGCTCCTCATCGAACTGCACCACGCGGAACCCGTGTCGGCCAAAGAGGAGGTGAAGGACGTCAAGGCCGAACCTCAAGCTGAAACAGAGGAGGCCGGGGAGGAGCCTGTTTAG | 876 |
|  | MRSLRQKLQIRNRLARECLAEFLGEYMLILMGTAAVAQVVTNFDQKGTYLSINIGYAAGVLFGIYASVGVSGAHLNPAVTFSLCVLGRFPWRKLPFYTISECLGSFVASATTFTLYYDAIHEFSGGNLTVRGPRGTAGIFATYPVEYLSVRNGFITEVIGTAVLLICVLSVGDAKNAGAPAFLQPLLISVSVLVIGAAMGANTGYAINPARDLGPRLFTFVAGWGTEVFKAGNGWWWIPIVAPLIGGVIGSLAYTLLIELHHAEPVSAKEEVKDVKAEPQAETEEAGEEPV | 291 |
| 6 | North pacific spiny dogfish (*Squalus suckleyi*) Aqp0 |  |
|  | ATGATGTGGGAGCTGAAATCTATCTCCTTTTGGAGGGCAGTCTTTGCAGAGTTTTTTGCCACCATGCTCTTCGTGTTTTTTGGACTGGGCTTTACCATGCGATGGTCTCCGGGCCCCGTGAACGTGCTGCTGGTTTCTCTGGGTTTTGGCTTTGTCCTGGCTGCTTTGGTGCAGGCGGTTGGACATGTCAGCGGGGCGCACCTCAACCCGGCCGTTACCTTCGCCTACCTGCTAGGGGCGCAGCTGAGCATGTTCCGCTGTGTAATGTACATCGCAGCTCAGCTCCTGGGGGCAGTGGCGGGGGCAGCTGTGCTGTATGGGGTCACCCCTCCCGCGGTACGGGGCAACCTGGGGCTCAACACGCTGCATGCTGGAGTGGGTCCTGGCCAGGCTACAGCTGTGGAGATTTTCCTTACTCTTCAGTTCGTTCTCTGTATTTTTGCCACAACAGACATTCGCAGAAATGGATTCATGGGCTCAGCAGCTGTGATCATTGGTTTTTCCCTCACTGTGGGTCACTTCTTCGGGCTGTATTACACAGGATGTGGAATGAACCCAGCCAGGTCCTTTGCACCAGCCGTGCTCACTAGAAACTTTGGAAATCACTGGGTGTACTGGGTAGGTCCTCTGATAGGAGGTGCTATAGCAGCACTGCTGTACGATTTTATCCTCTTCCCCAGGATGAGAGGTCTCTCAGAGAGACTGGCTATTCTTAAAGGAGCTCATCCCCCAGAAGCTGAGGGACAACAGGAACCGAGGAGCGATCCCATCGAACTCAAAACACAAGCCTTATAA | 795 |
|  | MMWELKSISFWRAVFAEFFATMLFVFFGLGFTMRWSPGPVNVLLVSLGFGFVLAALVQAVGHVSGAHLNPAVTFAYLLGAQLSMFRCVMYIAAQLLGAVAGAAVLYGVTPPAVRGNLGLNTLHAGVGPGQATAVEIFLTLQFVLCIFATTDIRRNGFMGSAAVIIGFSLTVGHFFGLYYTGCGMNPARSFAPAVLTRNFGNHWVYWVGPLIGGAIAALLYDFILFPRMRGLSERLAILKGAHPPEAEGQQEPRSDPIELKTQAL | 264 |
| 7 | North pacific spiny dogfish (*Squalus suckleyi*) Aqp1 |  |
|  | ATGGTCAGAGAAGTCCAGCGCAAGACCTTCTGGAGGGCTGTGCTCGCCGAGTTCTTGGGGGTGACCATCTTCGTCTTTCTCAGCATCGGGTCGGCCACCAAGTGGACGCCCAGCGGCTTCCCCGCCGACGTGGTGCAGATCGCCCTGACCTTCGGGCTGTCCATCGCCACCCTGGCCCAGAGCATCGGCCACATCAGCGGGGCGCACCTCAACCCGGCCGTCACCCTGGGGCTGCTGGTGGGCTGCCAGATCAGCGTGCTGCGGGCCGTCCTGTACATGATCTCCCAGCTGCTGGGCGCCGTGGCAGCCAGCGCCATCCTCTTCGGTGTCACCCCCAACTCCAGGAACGGGACCCTCGGAGTCAACGCGCTGGGAGAAGGGGTTACCCCGGGGCAGGGTCTGGGCGTTGAAATTATCATCACCTTCCAGCTCGTTCTCTGCGTCTTTGCGACCACAGATAAACGAAGGACGGATCTCTCCGGCTCCGGCCCTCTAGCCATTGGACTTTCGGTTGCTATTGGCCACTTAATGGCGATTGGCTTCACCGGATGTGGGATGAACCCTGCCCGTTCCTTCGGGCCAGCTGTCATTACCGGCAACTTCAAGGATCACTGGCTGTACTGGGTGGGCCCCATGATCGGAGGATTCGTCGCCGCTCTGCTCTACGACTTTGTGTTCACCACGCCGTTCCGCGACCTGTCCGAGCGGCTCAAGGTGCTGAACAGCGGGCAGACAGAAGAATACGATGTGGAAGGGGAGGGGGACAGTGCCAGGATGGAAATGAAGCCAAAGTAA | 795 |
|  | MVREVQRKTFWRAVLAEFLGVTIFVFLSIGSATKWTPSGFPADVVQIALTFGLSIATLAQSIGHISGAHLNPAVTLGLLVGCQISVLRAVLYMISQLLGAVAASAILFGVTPNSRNGTLGVNALGEGVTPGQGLGVEIIITFQLVLCVFATTDKRRTDLSGSGPLAIGLSVAIGHLMAIGFTGCGMNPARSFGPAVITGNFKDHWLYWVGPMIGGFVAALLYDFVFTTPFRDLSERLKVLNSGQTEEYDVEGEGDSARMEMKPK | 264 |
| 8 | North pacific spiny dogfish (*Squalus suckleyi*) Aqp3c1 |  |
|  | ATGGGAAAACAAAAAGAGATCCTCAGAAAAATGACAACGACACTGAAAGTCCGAAGTATTCTGGTGAAACAATGTCTTGCTGAATGTTTAGGAACTCTAATTCATACAATGCTTAGCTGTGGAGCAATAGCACAATTTACTCTCGGTTATGGTACACACAAAGAATTTTTGACGGTTACTTTTGCCATCGGATTTGCAGTAGCTCTGGGTATAATGGTAACTAGTAAAGTGTCAGGAGCTCACCTGAATCCTGCAGTGACCTTTGCTTTGTGCTTGCTTGCTTGTGAGCCTTGGTTAAAATTCCCCTTCTTCTTTTTGGCACAAACATTCGGTGCCTTTCTTGGATCAGGAATAATGTTTGGTTTGTATTACGATAAATTGTGGCATTATGGTAATAAACAGCTAACAGTAATGGGACCAAACTCTACTGCTGGAATATTTACTACTTATCCGCCAGAACATTTGAGTGCAGTCAGTGGCATTTTTGATCAGGCAATTGGGACGGCAGCTCTGATACTTTGTATCCTTATCATTGTGGATCCAATGAACAAGCCAGTGCCAACAGGACTGGAAGCCTTTACCATTGGCTTTGTGGTTCTGATAATTGGCTGGTCAATGGATTCAAATTCCCAGTACTCATTAAATCCTGCCAGAGATATTGGACCTCGCTTGTTTACTGCAATTGCTGGTTGGGGATCTGAAGTTTTCACTGCTGGAAACTATTGGTTTTGGATCCCACTTGTCAGCCCAATCATTGGTGCCATTTTTGGTGTTCTGATGTATCTATTCATTGTTGGATTGCGTGTTGAAGCAAGAAGCGGCTGCTCACCCAATGCAGAACAAAATGTAAAGTTAATGAGCCAGAAACCAAAGGGGAGGCGCTAA | 885 |
|  | MGKQKEILRKMTTTLKVRSILVKQCLAECLGTLIHTMLSCGAIAQFTLGYGTHKEFLTVTFAIGFAVALGIMVTSKVSGAHLNPAVTFALCLLACEPWLKFPFFFLAQTFGAFLGSGIMFGLYYDKLWHYGNKQLTVMGPNSTAGIFTTYPPEHLSAVSGIFDQAIGTAALILCILIIVDPMNKPVPTGLEAFTIGFVVLIIGWSMDSNSQYSLNPARDIGPRLFTAIAGWGSEVFTAGNYWFWIPLVSPIIGAIFGVLMYLFIVGLRVEARSGCSPNAEQNVKLMSQKPKGRR | 294 |
| 9 | North pacific spiny dogfish (*Squalus suckleyi*) Aqp3c2 |  |
|  | ATGGGAAAACAAAAAGCAATAATCAGAAAAATTGAAGATTCATTCAGAATAAGAAATCTGTTGGTAAGACAATGTCTTGCTGAATGTTTAGGAACGTTGATTCTTGTGTTGTTTGGCTGTGGAGCATTGGCACAAATGACCCTCAGTAGGGGTACACACGGACAGTTTTTGACTGTTAATTTTGCCTTCGGATTTGCAGTGATGCTCGGTGTACTCCTGGCTGGCCAAGTCTCAGGAGCGCACTTGAATCCTGCCGTGACCTTTGCTATGTGCTTACTTGCTCGCGAACCCTGGTTAAAATTTCCCCTTTACTCTTTGGCACAAATACTAGGCGGCTTCCTTGGATCTGGTATCATTTTCGGTTTGTATTTTGATGCCATGTGGGACTTTAGTGGTCAAAACAAACTGTTAATATATGGCCCCAATGCCACTGCTGGTATATTCGCTACATACCCATCTGTGCACTTAACTCCACTCAATGGCTTTTTTGATCAGCTGATTGGAACTGCCGCTCTCATAGTTTGCATCCTTAGCATTGTGGATAAATTCAATAACCCGGTGCCAAAGGGACTGGAGGCATTTACTGTTGGCTTTACCGTCCTGGTAATTGGCTTGTCTATGGGTTTCAACTCTGGGTATGCCGTGAACCCTGCCAGAGACTTTGGACCTCGTTTGTTTACATCATTGGCTGGCTGGGGAGCTGAGGTTTTCATTGCTGGAAACTACTGGTTTTGGATCCCTATTTTTGCCCCACTCCTTGGTTCTGTACTTGGTATTCTGGTATATCAGCTTATGATTGGAATACACCTCGAGCCCGAAAACCACAATTCACCCATTGGAGAAGAAAATGTAAAACTGGCTAATGTAAAATTAAGAGAAAGTTCCTGA | 888 |
|  | MGKQKAIIRKIEDSFRIRNLLVRQCLAECLGTLILVLFGCGALAQMTLSRGTHGQFLTVNFAFGFAVMLGVLLAGQVSGAHLNPAVTFAMCLLAREPWLKFPLYSLAQILGGFLGSGIIFGLYFDAMWDFSGQNKLLIYGPNATAGIFATYPSVHLTPLNGFFDQLIGTAALIVCILSIVDKFNNPVPKGLEAFTVGFTVLVIGLSMGFNSGYAVNPARDFGPRLFTSLAGWGAEVFIAGNYWFWIPIFAPLLGSVLGILVYQLMIGIHLEPENHNSPIGEENVKLANVKLRESS | 295 |
| 10 | North pacific spiny dogfish (*Squalus suckleyi*) Aqp4 |  |
|  | ATGACGGCATTCAAAGGGATCTGGACGCAGCAGTTCTGGAGAGCAGTTTCTGGGGAGTTCCTGGCGACGCTCATCTTTGTCCTTCTCAGCCTAGGTTCCACCATTGGCTGGAGCGGTGAAGGTGGACCTTTGGACATTGTCCTCATCTCTCTCTCCTTCGGACTCAGCATCGCGACCATGGTCCAGTGCTTTGGTCACATCAGTGGGGGTCACGTTAACCCTGTGGTAACCGCTGCCATGGTCTGTACCAGGAAGCTCAGCCTGGCCAAGGGATTCTTCTACATTCTCGCGCAATGTCTCGGTGCCATTGTAGGAGCAGGAATCCTCTACCTGATCACGCCGTCAGATGTTGTGGGCGGACTAGGCGTTACCATGATAAATGAAAAGCTTTCAGCGGGCCATGGTCTGTTGGTTGAACTGTTTATAACATTCCAGCTGGTGTTTACCATCTTTGCCACTTGTGACTCTAAACGTGATGACCTCAAGGGCTCGGCAGCTTTGGCGATTGGTCTCTCTGTCGTCATTGGACACATGTTTGCCATTAACTACACTGGCGCTAGTATGAACCCTGCTCGATCATTTGGACCTGCCGTTATCACAGGGAAATGGGAAAACCATTGGGTTTACTGGGTCGGTCCTATGATGGGTGGGATCATCGCCGCTGCCCTCTACGAATATCTGTTCTGCCCACACAGAGAACTGAAGCACCACTTTAAAGATATTTTCAAGTCTACGCAGCCTTCCGGGGACAAATACGCGGAGGGGGAGGACAATAGGAGCCAAGCCATTGAGTACGAGGACCTGGCTGTCAAAGCTGGAGGCTCTCAAGTGATTGATGTCGATCAGAATGAAGAAAAGAAGGAAAAAGATGCCACCAAAGAACTCCTGTCATCTGTATGA | 900 |
|  | MTAFKGIWTQQFWRAVSGEFLATLIFVLLSLGSTIGWSGEGGPLDIVLISLSFGLSIATMVQCFGHISGGHVNPVVTAAMVCTRKLSLAKGFFYILAQCLGAIVGAGILYLITPSDVVGGLGVTMINEKLSAGHGLLVELFITFQLVFTIFATCDSKRDDLKGSAALAIGLSVVIGHMFAINYTGASMNPARSFGPAVITGKWENHWVYWVGPMMGGIIAAALYEYLFCPHRELKHHFKDIFKSTQPSGDKYAEGEDNRSQAIEYEDLAVKAGGSQVIDVDQNEEKKEKDATKELLSSV | 299 |
| 11 | North pacific spiny dogfish (*Squalus suckleyi*) Aqp8 |  |
|  | ATGTCAGCGAAAGAAGGAGCGGACGACCCAGGCACCGAACCCTCTGAAATGCTCTCCAAATGCACGCTGTACGAGCGGTACCTCCAGCCCTGTGTGGTCGAGCTGCTGGGGTCAGCTCTCTTTATCTTCTTCGGCTGTTTATCTGTGGTGGAAAACGTGGAGGGAACGGGACGGCTCCAGCCCGCCTTGGCTCACGGTTTGGCGCTGGGGCTCATCATCGCTATCCTCGGGAATATTAGTGGTGGACATTTTAATCCAGCAGTGTCCTTAGGAGCATATTTAATTGGTGGACTTAATATTATTCTACTCATTCCCTATTGGACTGCACAGCTATGTGGTGGAATGATTGGAGCAGCACTCACAAAGGTTGTTACACAAGATGGAAACTTTTTCAATGCAACTGGAGCAGCATTCACAGCTATTCAAAAGGACGAACATGTTGGAAGAGCAATTATCTGTGAGATAATCACGACAACCTTTCTTGTACTAGCAGTCTGCATGGGAGCTATTAATCAGAAAAGCACAACTCCTTTGGCACCGTTCTGCATTGGGTTTACTGTTACAGTAAATATCTTGGCTAGTGGTGGTTTGTCTGGGGCATGTATGAATCCTGCCCGAGCTTTTGGACCAGCAGTGGTGGCCAACTACTGGGACTATCATTGGGTCTACTGGGTGGGACCAATGATTGGAGCAGTGGTAGTAGGAATTTTAATCAGAATTCTGCTTGGAGACAGGAAAATCCGTCTAATTCTGAAATGA | 759 |
|  | MSAKEGADDPGTEPSEMLSKCTLYERYLQPCVVELLGSALFIFFGCLSVVENVEGTGRLQPALAHGLALGLIIAILGNISGGHFNPAVSLGAYLIGGLNIILLIPYWTAQLCGGMIGAALTKVVTQDGNFFNATGAAFTAIQKDEHVGRAIICEIITTTFLVLAVCMGAINQKSTTPLAPFCIGFTVTVNILASGGLSGACMNPARAFGPAVVANYWDYHWVYWVGPMIGAVVVGILIRILLGDRKIRLILK | 252 |
| 12 | North pacific spiny dogfish (*Squalus suckleyi*) Aqp9 |  |
|  | ATGGAGCCGCAGATCCAGAAGAAGAGTCTGATAGACAGATGCAAATTAAAGAACCGCTTCTTAAAAGAAGGGCTGGCCGAATTTCTGGGAACATTCATATTGATTCTGTTTGGTTGTGGTTCACTCGCCCAATCAATACTGAGCAGAGGAGTAAGTGGTAATATACTGACCTCTTCCATTGGCTTTCCATTTGGAATTACCATAGCTGTTTATGCTACAATGGGAGTTTCAGGGGGACACCTGAATCCTGCGATTTCCCTTTCTATGTGTGTGCTGGGACGGCTTCAATGGTTGAAACTTCCTGTCTACTGTGTTGCTCAGCTTTTCGGAGCCTTTATCGGTTCAGCGGCAGTTTTTGGGCTTTATTACGATGCCTTCATGGCTTTCGATGACGGGAACTTGACAATAACCGGTCAAAATGCAACAGCGCAGATTTTCTCCTCTTATCCAAGTCCACATTTATCATTTGCAAATGGCTTTGCAGATCAGATTGTGGGCGCTGCTGCACTGCTCTTCTCTATTCTGGCCATTTTGGACTCTAAGAATGATGGTGTACCCAAAGGTTTGGAGCCAGTGGTGATTGGCCTGATCGTCATGGTTATCGGTGTTTCAATGGGCTACAACTGTGGTTGTCCCATAAACCCAGCTCGTGACCTTGGACCCCGGCTCTTTACAGCTGTGGCCGGATGGGGATTTGAGGTTTTCAGTGCGGGCGATGGCTGGTGGTGGGTTCCTGTCATCGCACCAATGATTGGAGGAATGCTCGGGACTTCCATTTATGTTCTGATAATTGAGCTGCACCACAAAGAGTCGATCCCAGAGGAGAGCCGCTGTATTCCAGAACCGCAAATCGAAGGGAAGGCAAAATATGAAATGATTTCAATACAGTGTAACAAATAA | 900 |
|  | MEPQIQKKSLIDRCKLKNRFLKEGLAEFLGTFILILFGCGSLAQSILSRGVSGNILTSSIGFPFGITIAVYATMGVSGGHLNPAISLSMCVLGRLQWLKLPVYCVAQLFGAFIGSAAVFGLYYDAFMAFDDGNLTITGQNATAQIFSSYPSPHLSFANGFADQIVGAAALLFSILAILDSKNDGVPKGLEPVVIGLIVMVIGVSMGYNCGCPINPARDLGPRLFTAVAGWGFEVFSAGDGWWWVPVIAPMIGGMLGTSIYVLIIELHHKESIPEESRCIPEPQIEGKAKYEMISIQCNK | 299 |
| 13 | North pacific spiny dogfish (*Squalus suckleyi*) Aqp10c1 |  |
|  | ATGAGGAGAGCAGAAACATTCGTCAACAAGCTGAAAGATGTCTTGCAGTTGAAGAACCATCTCTTCAGAGAATGTCTAGCCGAGTTTCTAGGAGTTTGCATGCTGATTTTGTTCGGGTGTGGCGCAGTAGCACAGATGGTGACAAGCCACAGAACTCGAGGGAAATTTCTGTCGGTCAACCTGGGATTCGGCCTTGGTGCGACCTTTGGCACCTACATCGCTGGCGGTATCTCGGGTGCCCATCTGAACCCAGCCGTCTCCTTCAGCTTGTGTTTGTTGGGAAGGTTTCAGTGGAGGAAGCTGCCATTCTACATGTTCTTCCAGACCCTTGGGGGCTTTGTTGGTGCTGCTATAGTCTACGGGGTTCATCACGATGGTATACACGCGGTTGATAACGGGACTCTGTCTGTCACTGGTCCGCGAGCAACTGCGTTCATTTTTGGCACATATCCCGCATCGTACCTCACGCTTTCGAACGGCTTCATTGACCAGTTGATCGGCACCGCCACCCTGCTCCTCTGCATCCTGGCGGTGATAGACTCCCAGAACTGCGGAGCCCCGAAGCCCTTGCAGCCCATCTTCATCGGTCTGTCGGTCGTTGGCATCGGGATGTCCATGGGCTCCAACTCTGGCTACGCCATCAACCCCGCCCGGGACTTCGGCCCCCGCTTGCTAACCCTTGCAGCTGGCTGGGGCACAGAGGTCTTCACGGCTGGAGGTGGGTGGTGGTGGATCCCCATCGTGGCGCCCATGGTTGGCGCCGTCCTCGGCTCCCTCACCTACGAACTCCTCGTCGAGTTCCACCACCTGGCGGCCGAGGACAACCTCGACGAGAAGGAGCTGAAAGAGCAAAGGCCGAAGAAGAAGGGCAGCCCGCCCGTGAGCCATAAGCCAAGCGAGGCGTGCCAGCACGAGGCGGAGTGCGGGGTCGAGCAGTTCGCCTTGGCGATGTGA | 954 |
|  | MRRAETFVNKLKDVLQLKNHLFRECLAEFLGVCMLILFGCGAVAQMVTSHRTRGKFLSVNLGFGLGATFGTYIAGGISGAHLNPAVSFSLCLLGRFQWRKLPFYMFFQTLGGFVGAAIVYGVHHDGIHAVDNGTLSVTGPRATAFIFGTYPASYLTLSNGFIDQLIGTATLLLCILAVIDSQNCGAPKPLQPIFIGLSVVGIGMSMGSNSGYAINPARDFGPRLLTLAAGWGTEVFTAGGGWWWIPIVAPMVGAVLGSLTYELLVEFHHLAAEDNLDEKELKEQRPKKKGSPPVSHKPSEACQHEAECGVEQFALAM | 317 |
| 14 | North pacific spiny dogfish (*Squalus suckleyi*) Aqp10c2 |  |
|  | ATGAAGGTGTTTCGACAAAAGATCCGGATTAAGAACCACCTGGTGCGAGAATGTCTGGCAGAGTTCATAGGCGTCCTCATGCTGATTTTGATGGGAACTTCGACAGTGGCACAGGTGGTAACAAACTTCGATCGCAAGGGAACGTACTTGTCTATTAACTTCGGCTATGCCATCGGCGTCCTGTTTGGCATTTACACATCCGTCGGGATATCAGGCGCTCACTTGAACCCTGCAGTGTCCTTAAGCCTGTGCGTGCTGGGCAGGTTCTCATGGAGGAAGCTGCCGTTCTACATCGTGTCCGATTGTCTGGGGGCCTTCTGCGGTGCCGCCACCACCTACTGCGTTTACTACGGTATGTGGCACAGCGGGAAAGGCAACGGGACTCTGACTGTCACCGGCCCCAGAGGAACGGCGGGTATCTTTGCCACGTACCCGGCAGAAAACCTCAGTATGCGTAATGCGTTCCTTACCGAGCTAATAGGCACTGCTGTTTTGCTGATCTGTATTCTGTGCATTGGGGACGACCAAAACGCAGGGGCTCCAGCTTTCTTGCGGCCCTCCCTTGTCGCTGTCGCGGTGTTCGTCATTGGCATGGCGCTGGGCGCCAACACCGGCTACGCCATTAACCCGGCAAGAGACCTCGGACCCCGACTGTTCACCTTCGTTGCTGGGTGGGGCACTGAGGTATTCACGGCCGGACATGGATATTGGTGGATCCCGATCGTAGCGCCAATACTCGGAGCTCTGATCGGCACCATGATATACGAAGTCCTCATCGAGTTTCATCACGGCGATCCCAAGCCACTCAAAGACGAGGCCGAGCCGGCCGAGGAGGCCAAGGAAACGGCGGAGAATCCTGTGTAG | 864 |
|  | MKVFRQKIRIKNHLVRECLAEFIGVLMLILMGTSTVAQVVTNFDRKGTYLSINFGYAIGVLFGIYTSVGISGAHLNPAVSLSLCVLGRFSWRKLPFYIVSDCLGAFCGAATTYCVYYGMWHSGKGNGTLTVTGPRGTAGIFATYPAENLSMRNAFLTELIGTAVLLICILCIGDDQNAGAPAFLRPSLVAVAVFVIGMALGANTGYAINPARDLGPRLFTFVAGWGTEVFTAGHGYWWIPIVAPILGALIGTMIYEVLIEFHHGDPKPLKDEAEPAEEAKETAENPV | 287 |
| 15 | North pacific spiny dogfish (*Squalus suckleyi*) Aqp11 |  |
|  | ATGGAGGACGTCCTTGTTTCGCTCGGTGTCCTGGCCGGCACGGTAACGGTGTGCCAGGTGCTGCGCCGGACGGCCAAGGAGATACTGTACCCGCGGCGGCGGGGGGGGCGCGGGGGAGGAGGGCCGCGGCCCAGCGGCTCGGGGACGGACCCCCTGGAGGTGGCGGTCGAGCTCTTCTCCACCCTGCAGCTCTGCGTCTGCACCCAGGAGCTGCAGCTGCTGGGTCGCTCGGGGCTGCTGCCCGGGCTGTACCCGGGGCTGGGCATCACCTACCTCATGACCCTGGTCCACCTCTTCACCTTCGGCGGCGCCACCTGCAACCCGGTGAGCTGCCTGGAGCAGTACCTCCGCGGGCAGAGCGGCGGGCGGGCGCTGGCTGCCAAGCTGCTGGCGCAGTTCGGGGCCGCCTCGGTGGCGCGCCGGCTTGTCGAGCTGGTCTGGTCCCTGGACATGTCGGACATGCACTGGCATCACCGCCAGCGCGGGTACGCGTGCACCTCCTCCCTGAACGCAGACGCCGGCAACGGGGTGGTCGCGGAGTTTTTCTGCGCCTTTGCTGTGAGAGCGGTCCTCTTCAGAATTCAGCACCTGGGGCAGAGACGCAAGGCGCACGCGGTGGCCGCTTTGATCACCTGCCTTGTATTTGCAGCTGGAGACCTGACGGGGGCTGTGTTTAACCCAGCGCTGGCCTATTCCATCACCTTCAACTGCAAAGGAAGCACGTTTCTCGAATACAGCTTTGTTTACTGGCTTGGACCCTTAATGGGTGCAATGACAGCTGTGATGCTGTTTGATGAAAAATCGGTTCCGCAAACGCAGGAGGCGAACGAGAAAAGAGACTGA | 843 |
|  | MEDVLVSLGVLAGTVTVCQVLRRTAKEILYPRRRGGRGGGGPRPSGSGTDPLEVAVELFSTLQLCVCTQELQLLGRSGLLPGLYPGLGITYLMTLVHLFTFGGATCNPVSCLEQYLRGQSGGRALAAKLLAQFGAASVARRLVELVWSLDMSDMHWHHRQRGYACTSSLNADAGNGVVAEFFCAFAVRAVLFRIQHLGQRRKAHAVAALITCLVFAAGDLTGAVFNPALAYSITFNCKGSTFLEYSFVYWLGPLMGAMTAVMLFDEKSVPQTQEANEKRD | 280 |
| 16 | North pacific spiny dogfish (*Squalus suckleyi*) Aqp12 |  |
|  | ATGGCTGGGCTCAATGTCTCCTTCGGGTATTTCTTTGCTGTTGTGGCTTTCTGTGAGGTGGTCAGGAGGATCTCCAAAAAGCTTTTGCCACTTAAGATCTACTCCACCTTCCTGGTGGAGCTGGCCTCCTGCTTCCAGCTGGGTGCCTGTTGGTTTGAGCTCAGGATGTTGGTCATTATCGGGCCGTGGGGAGGTGGGTTTGGAATGGATGTGGTTATGACTCTGCTGTTTCTCCTTTACTTGATCCACGAAGCCACGTTTGACGGAGCTGAGGCCAACCCGCTAGTCACCGTTCAGGAATTGCTCCGCTCCAACTCTCCTGTGGTCGCCAGCACTCTGAAGATCTTGGCCCAGTTTGGGGGGACACAGTTGGCCAAGGTAGTCGCAAAACTGTACTGGTCCTGGGAGCTGACAGATTTACACCTCATCCAGAACATGATGGCCATGGACTGCAGCTCAGCCATCCAGACATCAGTCAGCCAAGGAGCCTTTGTTGAAGCGGCTTGCACCTTCCTTTTCCACCTAGTTGTGATGAAGTTTGAGGGAGCAGCTTTTGGATACAGGATCCTGTCCAAAGCCCTAACCATCACTGCCCTGGTGCACGTAGCTGGCCCATATACTACAGCTCTGTTTAACCCAGCACTTGCATTCCCAGTCACATTCCACTGTTCAGGAAACACATTGTCAGAGTACATGATAGTCTACTGGCTCAGTCCATTCATTGCAACAATTCTGGCCATCTTTCTGTTCAATGGAAACATTCCTCTGCTTTTCTGCAAGAATCTCCTCTATTCCCAAAGGACAAAGTACAAAATTCCTAAAGGGAAATCAACACCAGATCCAGAAGAGAACAAGGCAGTCAACAGGCAAGGACAGGCGACTTCTGGAAGAGGGGCTCGGAGATGA | 906 |
|  | MAGLNVSFGYFFAVVAFCEVVRRISKKLLPLKIYSTFLVELASCFQLGACWFELRMLVIIGPWGGGFGMDVVMTLLFLLYLIHEATFDGAEANPLVTVQELLRSNSPVVASTLKILAQFGGTQLAKVVAKLYWSWELTDLHLIQNMMAMDCSSAIQTSVSQGAFVEAACTFLFHLVVMKFEGAAFGYRILSKALTITALVHVAGPYTTALFNPALAFPVTFHCSGNTLSEYMIVYWLSPFIATILAIFLFNGNIPLLFCKNLLYSQRTKYKIPKGKSTPDPEENKAVNRQGQATSGRGARR | 301 |
| 17 | North pacific spiny dogfish (*Squalus suckleyi*) Aqp14 |  |
|  | ATGTCCTTCCGAGAGGTAAGGAAAGGGCGCAGGTTCTGGCGCTCGGTCTTAGCAGAGCTGGTTGGTTCCCTGGTCTTGGTTTCGGTTATTTTGGGCGCCTCTGCCCCTGGACAGGAAGATGGAGTTCCAGTGCTGATGCAGGTGGCTGTAGCTGCTGGGTTTTCTGCTGTCAGCCTGATCCACTGTTTTGGAGAGATTAGCGGTGCTCAGGTAAACCCGGCTGTCACACTCGCATTCCTGTGCACCAGGAAACTGGATTTTCTCCAGTTTGTGTCCTATCTTTTGGCTCAGTGTCTTGGAGCTGTGATTGGATCGGGGATAATCTACATGTCACTGCCGATCAAGTCAACATCAAGGCATTTAGTCAACATGATCAACAAAGATGGAAATGCTGGCCAAGCACTTGCGATGGAGATAATTGCCACATTCCAGTTGGTTTTCACTATATTTGCTGTGGATGATCATCGGCGTAGGGAAGTGGGAGAACCTGGCAGTCTAGCAATAGCCTTCAGCCTGTCAGCAGGAATCCTGGCATCGGGTAAGTTCTCAGGAGGCAGCCTGAACCCCGCAAGATCCCTTGGACCAGCGGTAATCACCGGATTCTGGGAGCATCACTGGGTGTACTGGATTGGCCCTCTCCTTGGTGCTGTCCTCGGTGGCATTTCTTACGAGTTCTTTTTTGCCTCCAGCGCCTCCCAAGAAAAGCTCATTGCCTGTATCACCTGCAAAGATATCGAGATTGTGGAAACCGCCAGCGTGTCCCGTTCATCGTTACTGACGGTCACGCAGTCCGCAATGCGAGCGAAACAGACTGCAAAAGTGCAGGACCACAGTTAA | 837 |
|  | MSFREVRKGRRFWRSVLAELVGSLVLVSVILGASAPGQEDGVPVLMQVAVAAGFSAVSLIHCFGEISGAQVNPAVTLAFLCTRKLDFLQFVSYLLAQCLGAVIGSGIIYMSLPIKSTSRHLVNMINKDGNAGQALAMEIIATFQLVFTIFAVDDHRRREVGEPGSLAIAFSLSAGILASGKFSGGSLNPARSLGPAVITGFWEHHWVYWIGPLLGAVLGGISYEFFFASSASQEKLIACITCKDIEIVETASVSRSSLLTVTQSAMRAKQTAKVQDHS | 278 |
| 18 | North pacific spiny dogfish (*Squalus suckleyi*) Aqp15 |  |
|  | ATGAAGCAGCTGAAGCAAGAACTGCAGAGCGAGTCCTTCTGGAGAGCTCTGACTGCCGAATTCCTGGGCACCGCCGTCTTCGTGTTCTCCAGTGTGGGCAGTGCGCTGTGCTGGGCTGGGCTGTGCCCCGGCACCCTGCAGGTGGCTCTGGGCTTCGGCCTGGGAGTGGCCGCCGTCTCAGTGTTCACACAACGAGTGAGCGGTGGTCAACTCAACCCCGCCGTCAGCCTGGCCCTTCTCCTCGCCCTGCGCATCAGCCCGCTCCGGGCGCTGCTCTACATGGCCATGCAAGCGCTGGGCGCGATCGCAGCCTGCGCCCTGCTGTACGCGCTCACACCGCCCAGCGTCCGCGGAGACCTGGGGCTCAACCAGCCTTCTCCGGGAGTTACACAGACTCAGGCCCTGGGAGTAGAAATCATTGTCACCTTCCAGCTTGTACTGTGTGTCTTCGCCGTCTCACACAAGAAAAGCAACTTTGAAGGGTCTGCGCACGTGGCCCTTGGAGCATCCGTGACCCTTGGACATTTGGTGGCAATTGGATTTACCGGATGCAGCATGAATCCTGCTCGATCCTTGGGACCAGCTGTCATTACAACAAATTTCAGTCATCATTGGGTGAGTGAACTGTGA | 630 |
|  | MKQLKQELQSESFWRALTAEFLGTAVFVFSSVGSALCWAGLCPGTLQVALGFGLGVAAVSVFTQRVSGGQLNPAVSLALLLALRISPLRALLYMAMQALGAIAACALLYALTPPSVRGDLGLNQPSPGVTQTQALGVEIIVTFQLVLCVFAVSHKKSNFEGSAHVALGASVTLGHLVAIGFTGCSMNPARSLGPAVITTNFSHHWVSEL | 209 |

| **Table S3.** Analysis of the synteny of *aqp3* in the cartilaginous genome databases. | | | | | | | | | | | | | | |
| --- | --- | --- | --- | --- | --- | --- | --- | --- | --- | --- | --- | --- | --- | --- |
| No. | Species | Common name | Chr. | Direction | Synteny | Synteny | Synteny | Synteny | Synteny | Synteny | Synteny | Synteny | Synteny | Synteny |
| 1 | *Callorhinchus milii* | Elephant shark | NW_024704742.1 | Plus |  |  |  | >ubap2a XP_042191625.1 XM_042335691.1 | <ube2r2 XP_042191676.1 XM_042335742.1 | >aqp3c1 XP_007895330.1 XM_007897139.2 | >aqp3c2 XP_007895329.1 XM_007897138.1 | <tpgs2 XP_007895328.1 XM_007897137.2 | >kiaa1328 XP_007895327.1 XM_007897136.2 | <celf4 XP_042191717.1 XM_042335783.1 |
| 2 | *Carcharodon carcharias* | Great white shark | 1 | Plus |  |  | >ubap2a XP_041043368.1 XM_041187434.1 | <ube2r2 XP_041052404.1 XM_041196470.1 | >nol6 XP_041044920.1 XM_041188986.1 | >aqp3c1 NC_054467.1 201141694-201165858 | >aqp3c2 XP_041054166.1 XM_041198232.1 | <tpgs2 XP_041030001.1 XM_041174067.1 | >kiaa1328 XP_041029463.1 XM_041173529.1 | <celf4 XP_041047221.1 XM_041191287.1 |
| 3 | *Chiloscyllium plagiosum* | Whitespotted bambooshark | 1 | Plus |  |  | >ubap2a XP_043543586.1 XM_043687651.1 | <ube2r2 XP_043543636.1 XM_043687701.1 | >nol6 XP_043541946.1 XM_043686011.1 | >aqp3c1 XP_043541859.1 XM_043685924.1 | >aqp3c2 XP_043541616.1 XM_043685681.1 | <tpgs2 XP_043541366.1 XM_043685431.1 | >kiaa1328 XP_043540470.1 XM_043684535.1 | <celf4 XP_043540966.1 XM_043685031.1 |
| 4 | *Hemiscyllium ocellatum* | Epaulette shark | 1 | Minus |  |  | >ubap2a XP_060711811.1 XM_060855828.1 | <ube2r2 XP_060685572.1 XM_060829589.1 | >nol6 XP_060711362.1 XM_060855379.1 | >aqp3c1 XP_060692140.1 XM_060836157.1 | >aqp3c2 XP_060685563.1 XM_060829580.1 | <tpgs2 XP_060685545.1 XM_060829562.1 | >kiaa1328 XP_060685529.1 XM_060829546.1 | <celf4 XP_060685363.1 XM_060829380.1 |
| 5 | *Heptranchias perlo* | Sharpnose sevengill shark | 1 | Minus |  |  | >ubap2a XP_067840806.1 XM_067984705.1 | <ube2r2 XP_067840709.1 XM_067984608.1 | >nol6 XP_067840686.1 XM_067984585.1 | >aqp3c1 XP_067840664.1 XM_067984563.1 | >aqp3c2 XP_067840639.1 XM_067984538.1 | <tpgs2 XP_067840620.1 XM_067984519.1 | >kiaa1328 XP_067840584.1 XM_067984483.1 | <celf4 XP_067847874.1 XM_067991773.1 |
| 6 | *Squalus Suckleyi* | North pacific Spiny dogfish | JAOAMX010013878.1 | Plus |  |  | >ubap2a 2376143..2460645 | <ube2r2 2475654..2597824 | >nol6 2624992..2740689 | >aqp3c1 2774902.. 2799795 | >aqp3c2 2821261..2848049 | <tpgs2 2922657..2947483 | >kiaa1328 2984620..3185121 | <celf4 3271010..4155425 |
| 7 | *Homo sapiens* | Human | 9 | Minus |  | >ubap2 NP_001356991.2 NM_001370062.2 | <ube2r2 NP_060281.2 NM_017811.4 | … | >nol6 NP_075068.2 NM_022917.5 | >aqp3 NP_001305073.1 NM_001318144.2 | >aqp7 NP_001161.1 NM_001170.3 |  |  |  |
| 8 | *Mus musculus* | Mouse | 4 | Minus |  |  | >ubap2 XP_006538287.1 XM_006538224.2 | <ube2r2 NP_080551.1 NM_026275.4 | >nol6 NP_631982.2 NM_139236.4 | >aqp3 NP_057898.2 NM_016689.2 | >aqp7 NP_031499.1 NM_007473.4 |  |  |  |
| 9 | *Crocodylus porosus* | Australian saltwater crocodile | NW_017728909.1 | Minus |  |  | >ubap2 XP_019387395.1 XM_019531850.1 | <ube2r2 XP_019387400.1 XM_019531855.1 | >nol6 XP_019387388.1 XM_019531843.1 | >aqp3 XP_019387387.1 XM_019531842.1 | >aqp7 XP_019387385.1 XM_019531840.1 | <tpgs2 XP_019387383.1 XM_019531838.1 | >kiaa1328 XP_019387382.1 XM_019531837.1 | <celf4 XP_019387373.1 XM_019531828.1 |
| 10 | *Xenopus tropicalis* | Tropical clawed frog | 1 | Minus |  | >ubap2 XP_031750804.1 XM_031894944.1 | <ube2r2 NP_001072702.1 NM_001079234.1 | <DKFZp434B061 XP_031762654.1 XM_031906794.1 | >nol6 NP_001011358.1 NM_001011358.1 | >aqp3 NP_001016845.1 NM_001016845.2 | >aqp7 NP_001015726.1 NM_001015726.1 | <tpgs2 NP_001016890.1 NM_001016890.2 | >kiaa1328 XP_031750801.1 XM_031894941.1 | <celf4 XP_031757514.1 XM_031901654.1 |
| 11 | *Latimeria chalumnae* | Coelacanth | 1 | Plus |  |  |  | <ube2r2 XP_006004794.1 XM_006004732.3 | >nol6 XP_064421093.1 XM_064565023.1 | >aqp3 XP_006004793.1 XM_006004731.1 | >aqp7 XP_064409331.1 XM_064553261.1 | <tpgs2 XP_064409339.1 XM_064553269.1 | >kiaa1328 XP_005990617.1 XM_005990555.3 | <celf4 XP_064421267.1 XM_064565197.1 |
| 12 | *Lepisosteus oculatus* | Spotted gar | 3 | Plus |  | >celf4 XP_069043531.1 XM_069187430.1 | <kiaa1328 XP_015219104.2 XM_015363618.2 | >tpgs2 XP_069043546.1 XM_069187445.1 | … | <ubap2a XP_069043559.1 XM_069187458.1 | … | <ube2r2 XP_006626665.1 XM_006626602.3 | >nol6 XP_069043565.1 XM_069187464.1 | >aqp3 XP_006626763.1 XM_006626700.2 |
| 13 | *Danio rerio* | Zebrafish | 3 | Plus |  |  |  |  |  | >aqp3a NP_998633.1 NM_213468.1 |  |  |  |  |
|  |  |  | 21 | Plus | <ube2r2 XP_005161075.1 XM_005161018.5 | … | >ubap2a XP_017208298.1 XM_017352809.3 | … | >aqp7 XP_068071903.1 XM_068215802.1 | <tpgs2 NP_001002208.1 NM_001002208.1 | >zgc:162344 NP_001077305.1 NM_001083836.1 | <celf4 XP_068071937.1 XM_068215836.1 | ... | >aqp3b NP_001159593.1 NM_001166121.1 |

| **Table S4.** Analysis of the synteny of *aqp9* in the cartilaginous genome databases. | | | | | | | | | | | | | | | | |
| --- | --- | --- | --- | --- | --- | --- | --- | --- | --- | --- | --- | --- | --- | --- | --- | --- |
| No. | Species | Common name | Chr. | Direction | Synteny | Synteny | Synteny | Synteny | Synteny | Synteny | Synteny | Synteny | Synteny | Synteny | Synteny | Synteny |
| 1 | *Callorhinchus milii* | Elephant shark | NW_024704767.1 | Minus |  | >cgnl1 XP_042200000.1 XM_042344066.1 | >myzap XP_042199956.1 XM_042344022.1 | >aldh1a2 XP_007904660.1 XM_007906469.2 | >aqp9 XP_007904652.1 XM_007906461.2 | >lipca XP_007904648.1 XM_007906457.2 | <adam10a XP_042200063.1 XM_042344129.1 | >mnidy2 XP_007904636.2 XM_007906445.2 | <sltm XP_042200019.1 XM_042344085.1 | >rnf111 XP_042199992.1 XM_042344058.1 |  |  |
| 2 | *Carcharodon carcharias* | Great white shark | 26 | Plus | >cgnl1 XP_041030862.1 XM_041174928.1 | >myzap XP_041031047.1 XM_041175113.1 | >polr2m XP_041031050.1 XM_041175116.1 | <aldh1a2 XP_041031018.1 XM_041175084.1 | >aqp9 XP_041030986.1 XM_041175052.1 | >lipca XP_041030954.1 XM_041175020.1 | <adam10a XP_041030753.1 XM_041174819.1 | >mnidy2 XP_041030758.1 XM_041174824.1 | <sltm XP_041030764.1 XM_041174830.1 | >rnf111 XP_041031102.1 XM_041175168.1 |  |  |
| 3 | *Chiloscyllium plagiosum* | Whitespotted bambooshark | 36 | Minus | >cgnl1 XP_043533390.1 XM_043677455.1 | >myzap XP_043533389.1 XM_043677454.1 | >polr2m XP_043533001.1 XM_043677066.1 | <aldh1a2 XP_043533388.1 XM_043677453.1 | >aqp9 XP_043533387.1 XM_043677452.1 | >Lipca XP_043533385.1 XM_043677450.1 | <adam10a XP_043533384.1 XM_043677449.1 | >mnidy2 XP_043533383.1 XM_043677448.1 | <sltm XP_043533374.1 XM_043677439.1 | >rnf111 XP_043533380.1 XM_043677445.1 |  |  |
| 4 | *Hemiscyllium ocellatum* | Epaulette shark | 39 | Minus | >cgnl1 XP_060709013.1 XM_060853030.1 | >myzap XP_060708818.1 XM_060852835.1 | >polr2m XP_060708819.1 XM_060852836.1 | <aldh1a2 XP_060709255.1 XM_060853272.1 | >aqp9 XP_060709247.1 | >lipca XP_060708848.1 XM_060852865.1 | <adam10a XP_060708986.1 XM_060853003.1 | >mnidy2 XP_060708945.1 XM_060852962.1 | <sltm XP_060708987.1 XM_060853004.1 | >rnf111 XP_060709024.1 XM_060853041.1 |  |  |
| 5 | *Heptranchias perlo* | Sharpnose sevengill shark | 34 | Minus | >cgnl1 XP_067827612.1 XM_067971511.1 | >myzap XP_067827239.1 XM_067971138.1 |  | <aldh1a2 XP_067827609.1 XM_067971508.1 | >aqp9 XP_067827607.1 XM_067971506.1 | >LIPCa XP_067827606.1 XM_067971505.1 | <adam10a XP_067827601.1 XM_067971500.1 | >mnidy2 XP_067827604.1 XM_067971503.1 | <sltm XP_067827595.1 XM_067971494.1 | >rnf111 XP_067827598.1 XM_067971497.1 |  |  |
| 6 | *Squalus Suckleyi* | North pacific Spiny dogfish | JAOAMX010014365.1 | Minus | >cgnl1 14677844..14780836 | >myzap 14605118..14630704 | >polr2m 14582676..14585743 | <aldh1a2 14390641..14481310 | >aqp9 14292843..14313293 | >lipca 14182150..14219610 | <adam10a 14070790..14171247 | >mnidy2 13978739..14030329 | <sltm 13927472..13965246 | >rnf111 13796462..13896642 |  |  |
| 7 | *Homo sapiens* | Human | 15 | Plus | >cgnl1 NP_116255.2 NM_032866.5 | >myzap NP_001018110.1 NM_001018100.5 | >polr2m NP_056347.1 NM_015532.5 | <aldh1a2 NP_003879.2 NM_003888.4 | >aqp9 NP_001307564.1 NM_020980.5 | >lipc NP_000227.2 NM_000236.3 | <adam10 NP_001101.1 NM_001110.4 | >sltm NP_001035540.1 NM_001040450.3 | >rnf111 NP_060080.6 NM_017610.8 |  |  |  |
| 8 | *Mus musculus* | Mouse | 9 | Minus |  |  | >polr2m NP_848717.1 NM_178602.3 | <aldh1a2 NP_033048.2 NM_009022.4 | >aqp9 XP_006511395.1 NM_022026.3 | >lipc NP_001311402.1 NM_001324473.1 | <adam10 XP_030099887.1 XM_030244027.2 | >mnidy2 XP_011241038.1 XM_011242736.4 | <sltm XP_006511412.1 XM_006511349.5 | >rnf111 NP_001361682.1 NM_001374753.1 |  |  |
| 9 | *Crocodylus porosus* | Australian saltwater crocodile | NW_017728911.1 | Plus |  | >cgnl1 XP_019389037.1 XM_019533492.1 | >myzap XP_019388709.1 XM_019533164.1 | <aldh1a2 XP_019389034.1 XM_019533489.1 | … | >lipc XP_019389026.1 XM_019533481.1 | <adam10 XP_019389023.1 XM_019533478.1 | >fam63b XP_019389022.1 XM_019533477.1 | <sltm XP_019389019.1 XM_019533474.1 | >rnf111 XP_019389018.1 XM_019533473.1 |  |  |
| 10 | *Xenopus tropicalis* | Tropical clawed frog | 3 | Minus | >cgnl1 XP_017947908.2 XM_018092419.2 | >myzap NP_001107425.3 NM_001113953.3 | >polr2m NP_001192173.1 NM_001205244.1 | <aldh1a2 NP_001039196.1 NM_001045731.1 | >aqp9 XP_002937719.2 XM_002937673.5 | >ttc24 XP_031755031.1 XM_031899171.1 | >lipc XP_012825482.1 XM_012970028.3 | <adam10 NP_001037869.1 NM_001044404.1 | >mnidy2 XP_002937714.1 XM_002937668.5 | … | <rnf111 XP_012826026.2 XM_012970572.3 | >sltm XP_012825429.1 XM_012969975.2 |
| 11 | *Latimeria chalumnae* | Coelacanth | 13 | Minus |  | >cgnl1 XP_014345558.1 XM_014490072.2 | >polr2m XP_064421794.1 XM_064565724.1 | <aldh1a2 XP_005998797.2 XM_005998735.3 | >aqp9 XP_005998794.1 XM_005998732.3 | >ttc24 XP_014345553.1 XM_014490067.1 | >lipc XP_005998793.1 XM_005998731.3 | <adam10a XP_064421941.1 XM_064565871.1 | >mnidy2 XP_005998790.1 XM_005998728.3 | <sltm XP_005998788.1 XM_005998726.3 | >rnf111 XP_005998784.1 XM_005998722.3 |  |
| 12 | *Lepisosteus oculatus* | Spotted gar | 5 | Plus |  | >cgnl1 XP_015198766.2 XM_015343280.2 | >polr2m XP_015198789.2 XM_015343303.2 | <aldh1a2 XP_006628784.1 XM_006628721.3 | >aqp9 XP_015198748.1 XM_015343258.2 | >ttc24 XP_015198739.2 XM_015343253.2 | >lipc XP_015198848.1 XM_015343362.2 | <adam10a XP_006628787.3 XM_006628724.3 | >mnidy2 XP_015198676.1 XM_015343190.2 | <sltm XP_015198675.1 XM_015343189.2 | >rnf111 XP_069046633.1 XM_069190532.1 |  |
| 13 | *Danio rerio* | Zebrafish | 25 | Minus |  |  | <pus7 NP_001116170.1 NM_001122698.2 | >srpk2 XP_021326236.1 XM_021470561.2 | >aqp9a NP_001028268.1 NM_001033096.1 | <vwa5a XP_068073529.1 XM_068217428.1 | <adam10b XP_021326233.1 XM_021470558.2 |  |  |  |  |  |
|  |  |  | 7 | Plus |  |  | >Polr2m NP_001333109.1 NM_001346180.1 | <aldh1a2 NP_571925.1 NM_131850.1 | >aqp9b NP_001171215.1 NM_001177744.1 | >lipca NP_957316.1 NM_201022.1 | <adam10a XP_005168954.1 XM_005168897.5 | >mnidy2 NP_001004517.1 NM_001004517.1 | <sltm NP_998498.2 NM_213333.2 | <rnf111 XP_005168982.1 XM_005168925.5 |  |  |

| **Table S5.** Analysis of the synteny of *aqp8* in the cartilaginous genome databases. | | | | | | | | | | | | | |
| --- | --- | --- | --- | --- | --- | --- | --- | --- | --- | --- | --- | --- | --- |
| No. | Species | Common name | chr | Direction | synteny | synteny | synteny | synteny | synteny | synteny | synteny | synteny | synteny |
| 1 | *Callorhinchus milii* | Elephant shark | NW_024704757.1 | Minus | >slc5a11 XP_007891713.2 XM_007893522.2 | >tex47 XP_007891711.2 XM_007893520.2 | <arhgap17a XP_042196949.1 XM_042341015.1 | >lcmt1 XP_007891709.1 XM_007893518.2 |  |  |  |  |  |
| 2 | *Carcharodon carcharias* | Great white shark | 15 | Plus | >slc5a11 XP_041062487.1 XM_041206553.1 | >tex47 XP_041062867.1 XM_041206933.1 | <arhgap17a XP_041061588.1 XM_041205654.1 | >lcmt1 XP_041061596.1 XM_041205662.1 |  | >prkcba XP_041061600.1 XM_041205666.1 | >cacng3 XP_041061602.1 XM_041205668.1 | <HELZ XP_041062345.1 XM_041206411.1 | >rbbp6 XP_041062468.1 XM_041206534.1 |
| 3 | *Chiloscyllium plagiosum* | Whitespotted bambooshark | 21 | Minus | >slc5a11 XP_043566964.1 XM_043711029.1 | >tex47 XP_043567013.1 XM_043711078.1 | <arhgap17a XP_043567012.1 XM_043711077.1 | >lcmt1 XP_043567665.1 XM_043711730.1 |  | >prkcba XP_043567663.1 XM_043711728.1 | >cacng3 XP_043567011.1 XM_043711076.1 | <HELZ XP_043567661.1 XM_043711726.1 | >rbbp6 XP_043567660.1 XM_043711725.1 |
| 4 | *Hemiscyllium ocellatum* | Epaulette shark | 20 | Minus | >slc5a11 XP_060696970.1 XM_060840987.1 |  | <arhgap17a XP_060696746.1 XM_060840763.1 | >lcmt1 XP_060696262.1 XM_060840279.1 |  | >prkcba XP_060696740.1 XM_060840757.1 | >cacng3 XP_060696739.1 XM_060840756.1 | <HELZ XP_060696968.1 XM_060840985.1 | >rbbp6 XP_060696736.1 XM_060840753.1 |
| 5 | *Heptranchias perlo* | Sharpnose sevengill shark | 22 | Plus | >slc5a11 XP_067859100.1 XM_068002999.1 | >tex47 XP_067859101.1 XM_068003000.1 | <arhgap17a XP_067859106.1 XM_068003005.1 | >lcmt1 XP_067859110.1 XM_068003009.1 | >aqp8 XP_067859851.1 XM_068003750.1 | >prkcba XP_067859114.1 XM_068003013.1 | >cacng3 XP_067859116.1 XM_068003015.1 | <HELZ XP_067859118.1 XM_068003017.1 | >rbbp6 XP_067859117.1 XM_068003016.1 |
| 6 | *Squalus Suckleyi* | North pacific Spiny dogfish | JAOAMX010024751.1 | Minus | >slc5a11 697557-781376 | >tex47 670557-675553 | <arhgap17a 600093-637440 | >lcmt1 411434-461638 | >aqp8 377368.. 390452 | >prkcba 297582-41314 |  |  |  |
|  |  |  | JAOAMX010077607.1 | Plus |  |  |  |  |  |  | >cacng3 17038-38886 | <HELZ 56015-311594 | >rbbp6 326984-374588 |
| 7 | *Homo sapiens* | Human | 16 | Plus | >prkcb NP_002729.2 NM_002738.7 | >cacng3 NP_006530.1 NM_006539.4 | >rbbp6 NP_008841.2 NM_006910.5 | >TNRC6A NP_055309.2 NM_014494.4 | >slc5a11 NP_001339177.1 NM_001352248.3 | <arhgap17 NP_001006635.1 NM_001006634.3 | >lcmt1 NP_057393.2 NM_016309.3 | >aqp8 NP_001160.2 NM_001169.3 |  |
| 8 | *Mus musculus* | Mouse | 7 | Plus | >prkcb XP_030098077.1 XM_030242217.1 | >cacng3 NP_062303.2 NM_019430.2 | >rbbp6 XP_036008698.1 XM_036152805.1 | >TNRC6A XP_011240059.1 XM_011241757.1 | >slc5a11 XP_011240069.1 XM_011241767.4 | <arhgap17 XP_006508264.1 XM_006508201.4 | >lcmt1 XP_030098526.1 XM_030242666.1 | >aqp8 NP_031500.1 NM_001109045.1 |  |
| 9 | *Crocodylus porosus* | Australian saltwater crocodile | NW_017728927.1 | Minus | >slc5a11 XP_019397391.1 XM_019541846.1 | >tex47 XP_019397671.1 XM_019542126.1 | <arhgap17 XP_019397384.1 XM_019541839.1 | >lcmt1 XP_019397383.1 XM_019541838.1 | >aqp8 XP_019397382.1 XM_019541837.1 | >prkcb XP_019397670.1 XM_019542125.1 | >cacng3 XP_019397381.1 XM_019541836.1 | >rbbp6 XP_019397380.1 XM_019541835.1 |  |
| 10 | *Xenopus tropicalis* | Tropical clawed frog | 9 | Minus | <rbbp6 XP_031748649.1 XM_031892789.1 | >tex47 XP_031749540.1 XM_031893680.1 | <arhgap17 XP_004918093.1 XM_004918036.4 | >lcmt1 NP_001016033.1 NM_001016033.2 | >aqp8 NP_001107728.1 NM_001114256.1 | >prkcb XP_012825383.1 XM_012969929.3 | >cacng3 XP_002932480.1 XM_002932434.5 |  |  |
| 11 | *Latimeria chalumnae* | Coelacanth | 5 | Minus | >slc5a11 XP_005994835.2 XM_005994773.2 | >tex47 XP_014343129.1 XM_014487643.2 | <arhgap17a XP_064414788.1 XM_064558718.1 | >lcmt1 XP_005994819.1 XM_005994757.2 | >aqp8 XP_005994818.1 XM_005994756.3 | >prkcb XP_064414784.1 XM_064558714.1 | >cacng3 XP_005998539.1 XM_005998477.1 | >rbbp6 XP_014345418.1 XM_014489932.2 |  |
| 12 | *Lepisosteus oculatus* | Spotted gar | 19 | Plus | >slc5a11 XP_015215743.2 XM_015360257.2 | >tex47 XP_015215829.2 XM_015360343.2 | <arhgap17a XP_015215825.2 XM_015360339.2 | >lcmt1 XP_015215827.1 XM_015360341.2 | >aqp8a XP_015215587.2 XM_015360101.2 | >aqp8b XP_069036991.1 XM_069180890.1 | >prkcb XP_069037108.1 XM_069181007.1 | >cacng3a XP_015215554.1 XM_015360068.2 | >rbbp6 XP_069037192.1 XM_069181091.1 |
| 13 | *Danio rerio* | Zebrafish | 12 | Minus |  |  | <arhgap17a NP_001017592.2 NM_001017592.3 | >lcmt1 NP_001004645.1 NM_001004645.2 | >aqp8.1a NP_001004661.1 NM_001004661.1 | >aqp8.1b NP_001073651.1 NM_001080182.1 |  |  |  |
|  |  |  | 3 | Minus |  |  | >slc5a11 NP_001007301.1 NM_001007300.1 | <arhgap17b NP_001316796.1 NM_001329867.1 | >aqp8.2 NP_001108382.2  NM_001114910.3 |  |  |  |  |

**Table S6.** *P*-values from Dunn's multiple comparisons test for Figures 5.

| Dunn's multiple comparisons test | Adjusted *p*-value | | | |
| --- | --- | --- | --- | --- |
|  | Water | Glycerol | Urea | Boric acid |
| control vs. HpeAqp3c1 | >0.9999 | >0.9999 | >0.9999 | >0.9999 |
| control vs. HpeAqp3c2 | <0.0001 | 0.0001 | 0.0002 | 0.4619 |
| control vs. HpeAqp8 | <0.0001 | >0.9999 | 0.0007 | <0.0001 |
| control vs. HpeAqp9 | <0.0001 | <0.0001 | <0.0001 | <0.0001 |
| control vs. HpeAqp10c1 | <0.0001 | 0.0561 | >0.9999 | >0.9999 |
| control vs. HpeAqp10c2 | 0.0036 | <0.0001 | 0.0011 | 0.1887 |
| HpeAqp3c1 vs. HpeAqp3c2 | <0.0001 | 0.0407 | 0.0005 | 0.4983 |
| HpeAqp3c1 vs. HpeAqp8 | <0.0001 | >0.9999 | 0.0013 | 0.0002 |
| HpeAqp3c1 vs. HpeAqp9 | <0.0001 | <0.0001 | <0.0001 | <0.0001 |
| HpeAqp3c1 vs. HpeAqp10c1 | <0.0001 | >0.9999 | >0.9999 | >0.9999 |
| HpeAqp3c1 vs. HpeAqp10c2 | 0.086 | <0.0001 | 0.0035 | 0.2831 |
| HpeAqp3c2 vs. HpeAqp8 | >0.9999 | 0.0162 | >0.9999 | 0.5607 |
| HpeAqp3c2 vs. HpeAqp9 | >0.9999 | 0.6435 | 0.6842 | 0.0025 |
| HpeAqp3c2 vs. HpeAqp10c1 | >0.9999 | >0.9999 | 0.0469 | >0.9999 |
| HpeAqp3c2 vs. HpeAqp10c2 | 0.3997 | >0.9999 | >0.9999 | >0.9999 |
| HpeAqp8 vs. HpeAqp9 | >0.9999 | <0.0001 | 0.3696 | >0.9999 |
| HpeAqp8 vs. HpeAqp10c1 | >0.9999 | 0.6523 | 0.093 | 0.073 |
| HpeAqp8 vs. HpeAqp10c2 | 0.1439 | <0.0001 | >0.9999 | 0.1833 |
| HpeAqp9 vs. HpeAqp10c1 | >0.9999 | 0.0117 | <0.0001 | 0.0001 |
| HpeAqp9 vs. HpeAqp10c2 | 0.4628 | >0.9999 | 0.0062 | <0.0001 |
| HpeAqp10c1 vs. HpeAqp10c2 | 0.0443 | 0.1577 | 0.3019 | >0.9999 |

**Table S7.** *P*-values from Dunn's multiple comparisons test for Figures 6.

| Dunn's multiple comparisons test | Adjusted *p*-value | | | |
| --- | --- | --- | --- | --- |
|  | Water | Glycerol | Urea | Boric acid |
| control vs. HpeAqp3c1 | >0.9999 | >0.9999 | >0.9999 | >0.9999 |
| control vs. CmiAqp3c1 | >0.9999 | >0.9999 | >0.9999 | >0.9999 |
| control vs. CplAqp3c1 | >0.9999 | >0.9999 | >0.9999 | >0.9999 |
| control vs. HpeAqp3c2 | <0.0001 | <0.0001 | 0.0003 | 0.1088 |
| control vs. CmiAqp3c2 | <0.0001 | <0.0001 | 0.0003 | 0.0105 |
| control vs. CplAqp3c2 | <0.0001 | 0.0004 | 0.0661 | >0.9999 |
| HpeAqp3c1 vs. CmiAqp3c1 | >0.9999 | >0.9999 | >0.9999 | >0.9999 |
| HpeAqp3c1 vs. CplAqp3c1 | >0.9999 | >0.9999 | >0.9999 | >0.9999 |
| HpeAqp3c1 vs. HpeAqp3c2 | 0.002 | 0.0199 | 0.0006 | 0.1078 |
| HpeAqp3c1 vs. CmiAqp3c2 | 0.0004 | 0.0008 | 0.0004 | 0.0143 |
| HpeAqp3c1 vs. CplAqp3c2 | 0.0002 | 0.2221 | 0.0591 | >0.9999 |
| CmiAqp3c1 vs. CplAqp3c1 | >0.9999 | >0.9999 | >0.9999 | >0.9999 |
| CmiAqp3c1 vs. HpeAqp3c2 | 0.0133 | 0.0041 | <0.0001 | 0.0239 |
| CmiAqp3c1 vs. CmiAqp3c2 | 0.0032 | 0.0001 | <0.0001 | 0.0026 |
| CmiAqp3c1 vs. CplAqp3c2 | 0.0016 | 0.0541 | 0.0026 | 0.3902 |
| CplAqp3c1 vs. HpeAqp3c2 | 0.004 | <0.0001 | 0.0002 | 0.0023 |
| CplAqp3c1 vs. CmiAqp3c2 | 0.0009 | <0.0001 | 0.0001 | 0.0002 |
| CplAqp3c1 vs. CplAqp3c2 | 0.0004 | 0.0011 | 0.0215 | 0.0671 |
| HpeAqp3c2 vs. CmiAqp3c2 | >0.9999 | >0.9999 | >0.9999 | >0.9999 |
| HpeAqp3c2 vs. CplAqp3c2 | >0.9999 | >0.9999 | >0.9999 | >0.9999 |
| CmiAqp3c2 vs. CplAqp3c2 | >0.9999 | >0.9999 | >0.9999 | >0.9999 |
